# Synthesis of [^13^C_3_]-B6 Vitamers Labelled at Three Consecutive Positions Starting from [^13^C_3_]-Propionic Acid

**DOI:** 10.3390/molecules23092117

**Published:** 2018-08-23

**Authors:** Thomas Bachmann, Michael Rychlik

**Affiliations:** Analytical Food Chemistry, Technische Universität München, Maximus-von-Imhof-Forum 2, D-85354 Freising, Germany; thomas.bachmann@tum.de

**Keywords:** labelled vitamers, pyridoxine, pyridoxal, pyridoxamine, vitamin B6, synthesis, isotopologues

## Abstract

[^13^C_3_]-labelled vitamers (PN, PL and PM) of the B6 group were prepared starting from [^13^C_3_]-propionic acid. [^13^C_3_]-PN was synthesized in ten linear steps with an overall yield of 17%. Hereby, higher alkyl homologues of involved esters showed a positive impact on the reaction outcome of the intermediates in the chosen synthetic route. Oxidation of [^13^C_3_]-PN to [^13^C_3_]-PL was undertaken using potassium permanganate and methylamine followed by acid hydrolysis of the imine derivative. [^13^C_3_]-PM could be prepared from the oxime derivative of [^13^C_3_]-PN by hydrogenation with palladium.

## 1. Introduction

**Figure 1:**
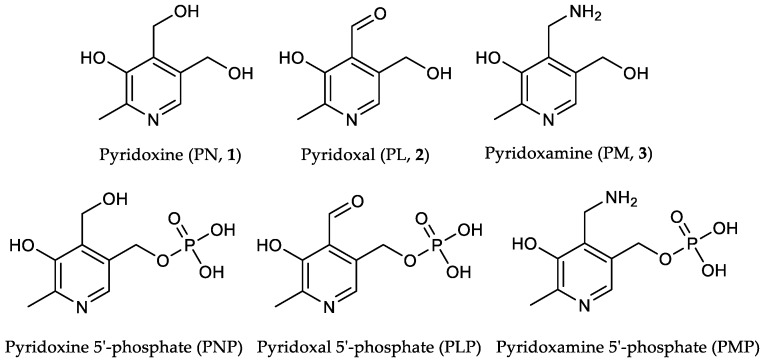
The term “vitamin B6” refers to six substrates sharing a 2-methyl 3-hydroxypyridine structure [1,2,3,4].

The group of vitamin B6 unites the water-soluble vitamers pyridoxine (PN, **1**), pyridoxal (PL, **2**), pyridoxamine (PM, **3**) and their respective phosphorylated derivatives pyridoxine 5′-phosphate (PNP), pyridoxal 5′-phosphate (PLP) and pyridoxamine 5′-phosphate (PMP) [1,2,3,4] (Figure 1).

Among these, PLP exhibits the highest biological activity and can be provided through in vivo transformation from the other aforementioned vitamers, which allows the enzymatically catalyzed biosynthesis of this vitamin as one of the main cofactors in human metabolism. In this regard, vitamin B6 participates in more than 160 enzymatic reactions, from which a major part includes amino acid biosynthesis and degradation [5,6,7,8,9].

First studies regarding this set of molecules blossomed in connection with the discovery of its function as antineuritic factor back in 1932 [10]. Because of its participation in a manifold of enzymatic reactions, the B6 group currently found its way into a various research fields, but nevertheless is mostly topical in medical research. Its correlation was examined inter alia to Parkinson’s disease [11], rheumatoid arthritis [12], depression [13], schizophrenia [14] antibacterial activity [15] and cancer [16,17]. Its potential as an antioxidant thus increasing resistance towards biotic and abiotic stress was also a matter of discussion [18]. Pyridoxine and its vitamers attracted further attention as it has been found to inhibit starch hydrolyzing enzymes in the GI tract and, therefore, may be potentially active in prevention of type2 diabetes [19].

**Scheme 1:**
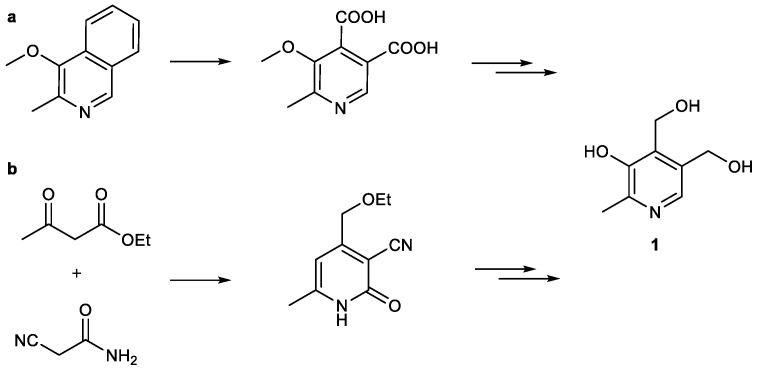
First preparations of pyridoxine (PN) utilized either degradation of (**a**) quinoline derivatives or (**b**) Knoevenagel reaction [20,21,22,23].

Synthetic approaches towards this group of vitamers, and in this regard specifically PN, started in 1939, on the one hand, by investigating a pyridone-condensation (b) from ethyl acetylacetate and cyanoacetamide as starting materials and, on the other hand, via oxidative degradation of isoquinoline derivatives (a) [20,21,22,23] (Scheme 1).

Other retrosynthetic strategies described the condensation of *N*-alkyl- or *N*-arylalkyl-alanine esters with α-formylsuccinic esters followed by *Dieckmann-cyclisation* or the assembly of the PN backbone from β-aminocrotononitril and ethyl hydroxymethyleneoxalacetate [24,25].

**Scheme 2:**
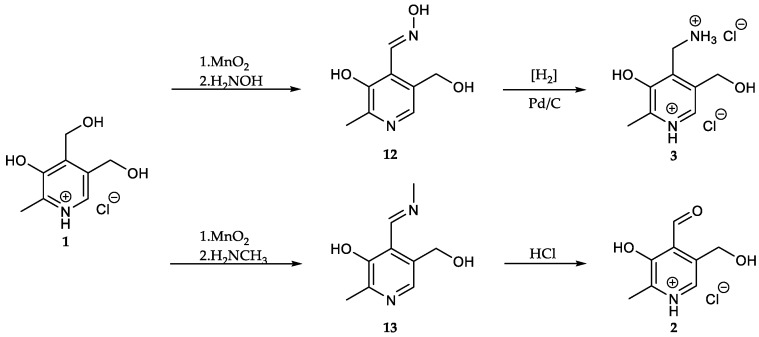
The chemical synthesis of pyridoxal (PL) and pyridoxamine (PM) is commonly furnished via imine derivatives and following hydrolysis/hydrogenation [31,32,33].

In 1944, studies regarding the influence of PN on the growth of lactic acid bacteria encouraged the search for further PN-based derivatives, which were later characterized as PL (**2**), PM (**3**) [26,27] and their respective phosphates [28,29,30]. Their synthesis commonly involves the formation of an imine derivative prepared by oxidation of PN (**1**) using manganese compounds in various oxidation states (i.e., KMnO_4_, MnO_2_) followed by reaction with amine substrates. Afterwards, acid hydrolysis or hydrogenation using palladium/platinum as catalyst leads to the desired product **2**/**3** [28,31,32,33] (Scheme 2).

**Scheme 3:**
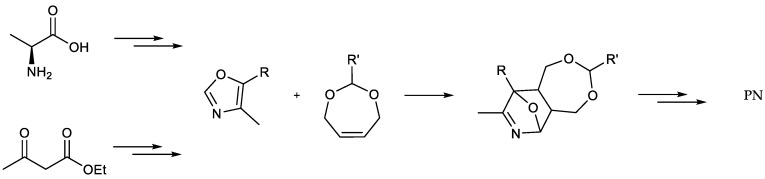
Implementation of Diels-Alder reactions into the synthetic strategies in the preparation of PN (**1**). (R = Alkyl, R′ = Isoalkyl/Alkyl).

A revolutionizing step enabling industrial scale production of PN involved the incorporation of *Diels-Alder* reactions into the synthetic route through condensation of 4,5-substituted-oxazoles and diverse ene-substrates in order to synthesize pyridine-based structures [34,35,36,37,38] (Scheme 3).

**Figure 2:**
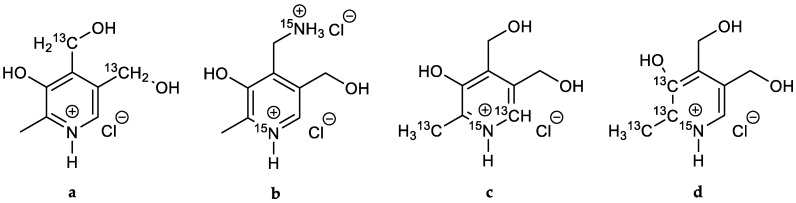
B6 vitamers (**a**–**d**) with different isotopically labelled positions/ atoms reported in the literature [31,32,43,44,45,46,47,48].

Building upon this strategic pathway, various modifications regarding the synthesis of PN have been developed until today [39,40,41,42], not only optimizing its preparation, but furthermore facilitating the implementation of isotopic labelling into the chemistry of vitamin B6. In that regard, carbon-^13^C labelling of the CH_2_-side chains in 4′ and 5′-positions has been reported using diethyl-di-^13^C-maleate ester (a) (Figure 2) or by acid catalyzed cyclisation of [1,4-^13^C_2_]-2-butenedinitrile and [1-^13^C,2-^15^N]-2-formylaminopropanenitrile [32,43,44,45]. In addition, nitrogen-^15^N isotopes have been integrated either inside the heterocycle or—in the case of amine derivatives—in the 4′-side chain (b) [31,46]. ^13^C- and ^15^N-labelling inside the core-structure was accomplished starting from either [^15^N-3-^13^C]-alanine and [^13^C]-formic acid (c) [47] or [^15^N-^13^C_3_]-alanine (d) [48], respectively.

**Scheme 4:**
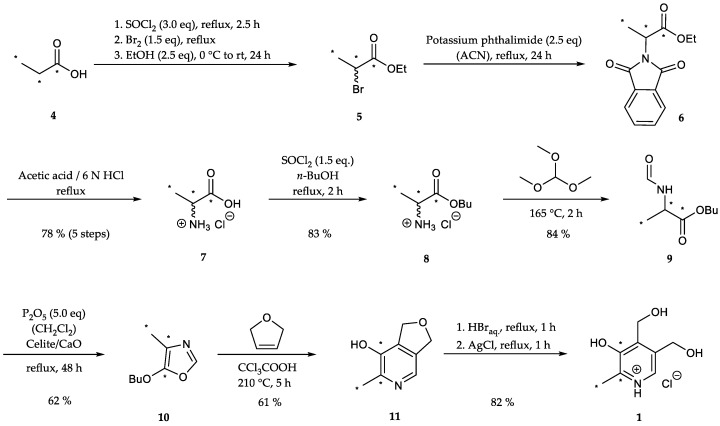
Reaction pathway for the synthesis of ^13^C_3_-PN starting from ^13^C_3_-propionic acid. (* = ^13^C).

Stable isotopologues of vitamins are particularly valuable as tracers in metabolic studies or as internal standards in quantitations by stable isotope dilution assays (SIDAs) [49]. In this regard, different labellings are of particular interest, when double isotope studies for differentiating between vitamers or between their function as tracer and internal standards have to be developed [50]. Although there exists a variety of synthetic strategies towards the preparation of stable isotopologues of the single vitamers, so far no preparation of [^13^C_3_]-labelled B6 vitamers starting from propionic acid has been reported. In pursuit of this purpose, the aim of this work focused on the investigation of current synthetic strategies in order to incorporate them into the preparation of isotopically labelled B6 vitamers. In order to guarantee a sufficient extent of labelling, which constitutes an important aspect regarding the application in SIDA inter alia because of the facilitated differentiation from other isotopologues, a consecutive threefold labelling within propionic acid has been chosen as starting material because of the direct incorporation into the “backbone” of PN when transformed according to the chosen reaction pathway (Scheme 4). Additionally, the commercial availability in proportion to its price played a decisive role when contrasted to other starting materials (e.g., alanine), which are frequently used in the literature.

## 2. Results and Discussion

### 2.1. Synthesis of ^13^C_3_-PN

As for the synthetic strategy, first, propionic acid was converted into alanine via halogenation and amination (Scheme 4). The second half of the reaction procedure comprised the conversion of the amino acid into PN through the formation of an oxazole intermediate followed by a Diels-Alder reaction.

Starting from propionic acid (**4**), halogenation in α-position was accomplished using thionyl chloride (SOCl_2_) and bromine (Br_2_) [51] (Scheme 4).

**Scheme 5:**
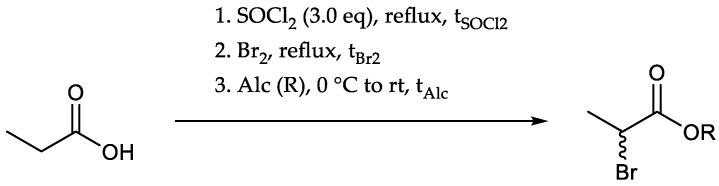
Preparation of the α-brominated propionic esters starting from propionic acid.

The in situ generated α-halogenated acyl halides were directly transformed into the respective esters with moderate to good yields (67–88%, Table 1, Scheme 5).

Variations of the reaction conditions indicated that aspects like brominating time (t_Br2_; 2d ~ 5.5 h > 3.5 h, Table 1, entry 1/2/3) and the scale of the reaction (PA; 5 g > 2 g > 1 g, Table 1, entry 2/4/7) had a higher impact on the reaction outcome, whereas the amount of added bromine (1.05/1.50 eq., Table 1, entry 2/3) or alcohol (Alc; 2.5–13 eq.) depicted only a minor influence. Alteration of the alcohol (R) showed that ethanol served well as esterification substrate, whereas methanol and *n*-butanol delivered lower yields (Table 1, entry 8/10). Using *tert*-butanol, no product could be isolated probably due to instability of the product under given conditions (Table 1, entry 9). Raising the temperature during the esterification step led to lower yields and increased formation of side products (Table 1, entry 6). Attempts to omit the esterification by addition of water instead of alcohol and thus to isolate the α-bromo carboxylic acid resulted also in lower yields (26%), whereas saponification of the α-halogenated esters led to the respective carboxylic acid in moderate yield (77%). Furthermore, the formation of a side product was observed independent of the reaction conditions (e.g., added bromine or reaction time). Although this substance can be separated from the product through distillation, we decided to skip this purification step and to immediately subdue the mixture to the next reaction because of potentially occurring losses of the target **6** during isolation.

The transformation of halogenated substrates into the respective amines can be carried out with various reagents like ammonia, ammonium chloride or phthalimide derivatives [31,52,53,54]. In this work, the selective amination in α-position was conducted applying the Gabriel synthesis. At first, potassium phthalimide, prepared from phthalimide and potassium hydroxide, was used to generate the *N*-substituted phthalimide **6** ([(M + H)^+^] *m/z* = 251.3). Subsequent attempts to cleave the phthalimide group with ethylene diamine or hydrazine hydrate—reagents which are frequently used for the mild removal of phthalimide groups [55,56,57,58,59,60,61]—led to the formation of undesired amide derivatives of the starting material irrespective of the reaction conditions. In order to circumvent this problem, we decided to cleave the phthalimide group through acid hydrolysis and remove the formed phthalic anhydride via extraction with ethyl acetate resulting in the hydrochloride salt of alanine **7** ([(M − HCl + H)^+^] *m/z* = 93.0) with an overall yield of 78% over the first five steps.

Because of the accompanying hydrolysis of the ester functionality during the removal of the phthalimide group, the next step involved the esterification with thionyl chloride and the respective alcohol resulting in the alanine butyl ester **8** ([(M − HCl + H)^+^] *m/z* = 149.0, 83%) [62].

**Scheme 6:**
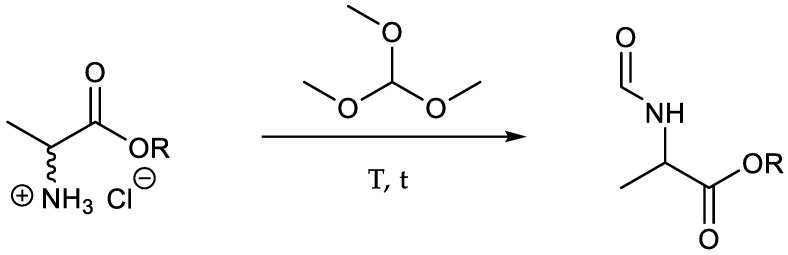
N-formylation was accomplished with trimethylorthoformiate (TMO).

*N*-formylation of amines can be accomplished according to a numerous selection of methods comprising different reagents, with one of the more often chosen being formic acid along with different additives in combination with acid or organic/metal catalysts [63,64]. A rather elegant method involves the use of triethylorthoformiate (TEO) [65,66,67]. Whereas the latter reagent is more commonly preferred, we chose the methyl derivate trimethylorthoformiate (TMO, Scheme 6) because of its lower boiling point, thus facilitating purification and resulting in the desired formylated product **9** ([(M + H)^+^] *m/z* = 177.2, 84%, Table 2, entry 16).

Main aspects affecting the outcome of the reaction besides reaction temperature and time (Table 2; entries 1–5) were also the size of the alkyl chain. Generally, higher yields could be achieved by either raising the temperature to 160 °C or maintaining the reaction time up to two hours. Choosing higher alkyl homologues increased the overall yield of the reaction almost regardless of the other parameters. This is illustrated by switching from an ethyl substituent to *n*-propyl and *n*-butyl, respectively. With the latter, reactions proceeded nearly quantitatively even at temperatures of 150 °C and reaction times of 1.5 h (Table 2; entries 6–15).

5-Alkoxy-4-methyl-oxazoles represent the key substrates of this synthetic path featuring their diene functionality. The ring closure is commonly undertaken by reaction with phosphorus pentoxide while sometimes adding oxides of alkaline earth metals [31,43,66,67,68]. Although a variety of derivatives have been investigated as substrate for Diels-Alder reactions, the reaction still remains the bottleneck of the pathway because of the rather moderate yield, mainly because of the volatility of the oxazole and its sensitivity towards hydrolysis.

**Scheme 7:**
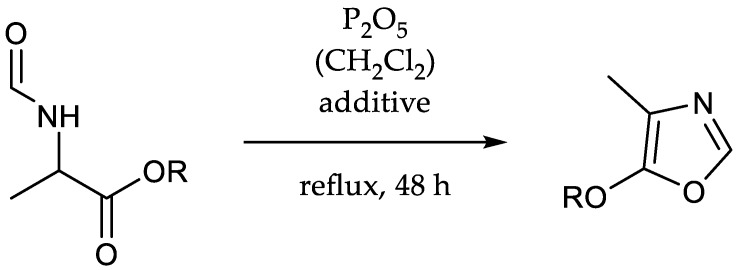
The cyclisation reaction was tested with various additives.

In this work, we focused on the impact of the addition of alkoxides and the variation of the alkyl chain on the reaction outcome (Scheme 7). First, the effect of magnesium oxide (MgO) without the use of celite was examined. This attempt resulted in a rather unsatisfying outcome (22%, Table 3, entry 1).

Increasing the amount of MgO, dividing the addition of phosphorus pentoxide (P_2_O_5_) or using celite, on the one hand, did not affect the yield to a great extent (27%, Table 3, entry 2), while utilizing a higher excess of MgO, on the other hand, led to a significant increase (40%, Table 3, entry 3). Switching to tin chloride (SnCl_2_) or no addition of an additive (not shown) led to worse or no reaction compared to MgO (19%, Table 3, entry 4). Compared to the previous experiments, switching to calcium oxide (CaO) resulted in an improved yield right away (46–48%, Table 3, entry 5/6). Furthermore, exchanging the alkoxy-substituent by a higher homologue resulted in higher yields (48–70%, Table 3, entry 6/7/8). This is probably due to, inter alia, the resulting lower volatility of the product and, therefore, presumably lower losses during the isolation of the product. While the preparation of these oxazoles (e.g., 5-butoxy-oxazole) was reported starting from other substrates such as *n*-butyl α-isocyanopropionate or 1-bromo-1-*n*-butoxyacetone, the influence of the alkoxy chain on this specific cyclisation procedure using P_2_O_5_ has not been documented in the literature yet [38,42]. The labelled oxazole **10** was finally prepared using the optimized reaction conditions and verified via ESI-MS ([(M + H)^+^] *m/z* = 159.1, 62%, Table 3, entry 9).

**Scheme 8:**
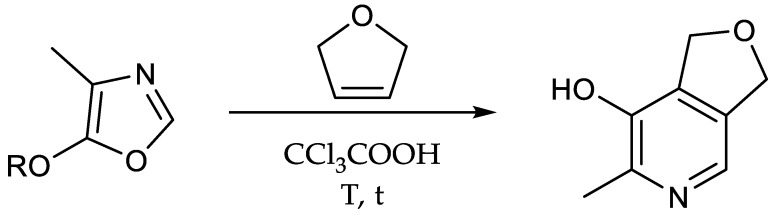
The pyridoxine-core was furnished via Diels-Alder reaction.

Utilizing the diene structure of the oxazole, the transformation of **10** involved a Diels-Alder reaction with 2,5-dihydrofuran (2,5-DHF) in a pressure vial to obtain the bicyclic compound **11** ([(M + H)^+^] *m/z* = 155.1, 61%, Table 4, entry 4) [31]. In accordance with earlier reaction steps during this study a longer alkyl chain suggested a higher yield (57–72%, Scheme 8, Table 4, entry 1/3). Furthermore, the absence of trichloroacetic acid (CCl_3_COOH) resulted in a drastically lower reaction outcome (27%, Table 4, entry 2).

The subsequent ring opening by hydrolysis leading to the bromine salt of pyridoxine was performed using aqueous hydrogen bromide. Finally, the hydrochloride salt of PN (**1**) was obtained by reaction with silver chloride ([(M − HCl + H)^+^] *m/z* = 173.0, 82%) [21,22].

Generally, all synthesized products were verified via ESI-MS and NMR-spectroscopy. In the latter, the signals of the labelled carbon atoms in the respective ^13^C-NMRs showed the same shifts as unlabeled substrates, but differed with regard to the resulting multiplicities (e.g., methyl-^13^C splitting up into a doublet due to interaction with the nearby ^13^C-carbon) and overall intensity.

As mentioned in the introduction, the preparation of fourfold-isotopically labelled PN was reported in the literature in five steps with a yield of approximately 25% [48]. The major difference to our synthetic route is that we added five reaction steps dedicated to the preparation of [^13^C_3_]-labelled alanine. Therefore, we increased the total linear steps to ten, while obtaining PN in an overall yield of 17%. Hereby, Caulkins et al. chose [^15^N-^13^C_3_]-alanine as a starting point, whereas [^13^C_3_]-propionic acid marked ours. Both strategies intertwine in alanine being a substrate, but our proposed route holds the potential to include also a ^15^N-nitrogen when using ^15^N-ammonia labelled substrates (e.g., ^15^N-phtalimide) resulting in the same substrate. Here, as a further aspect, the factor of costs has to be taken into account: although both substrates being commercially available, fully labelled alanine represents a rather expensive investment, whereas the use of fully labelled propionic acid and the fact that alanine can be prepared in the first five steps in 78% yield, grants a higher return regarding the amount of finally produced substrate even if ^15^N-labelled substances are included into the account for additional labelling. Moreover, as both strategies make use of a Diels-Alder reaction, further isotopes could be incorporated with isotopically labelled ene-substrates.

### 2.2. Synthesis of [^13^C_3_]-PL and [^13^C_3_]-PM

**Scheme 9:**
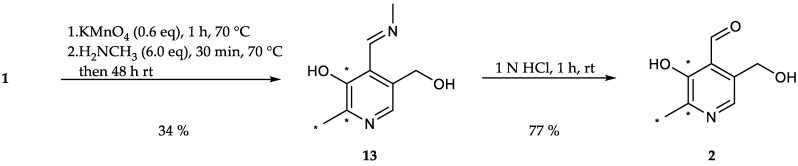
The route towards [^13^C_3_]-PL (**2**) led via the imine derivative **13** of [^13^C_3_]-PN (**1**).

With [^13^C_3_]-PN in hand, the path towards other vitamers of the B6 group started with the preparation of [^13^C_3_]-PL. Therefore, the primary alcohol in 4′-position was oxidized and directly transformed into the imine **13** by addition of methylamine hydrochloride ([(M + H)^+^] *m/z* = 184.0, 34%, Scheme 9).

While a diverse selection of procedures has been reported regarding the oxidation of PN, most reactions have in common the utilization of manganese dioxide (MnO_2_) as oxidizing reagent [69,70]. With water as solvent [31,32,33], the implementation of MnO_2_, either commercially available or freshly prepared colloidal one [71], resulted in mediocre yields (34%, Table 5, entry 1). Changing the solvent from water to toluene had a minor improving impact on the reaction (42%, Table 5, entry 2), while conversion of PN in toluene under removal of water through azeotropic distillation [72] resulted in the formation of various side products. Next to manganese dioxide, potassium permanganate (KMnO_4_) was also tested regarding the oxidation of PN [27], but did not improve the reaction outcome despite variation of the reaction conditions, e.g., variation of the amount of KMnO_4_ (0.4–1.6 eq.) *resp.* methylamine (CH_3_NH_2_) (3–10 eq.), reaction temperature (rt to 70 °C) and time or neutralization of PN hydrochloride beforehand (11–42%, Table 5, entries 3–12).

Substitution of methylamine with benzylamine or ethylene diamine in order to test other amine substrates did not provide the respective imine derivative. Efforts to prepare PL using TEMPO or IBX resulted in no transformation of the starting material at all. The obtained isolated imine was converted into [^13^C_3_]-PL (**2**) by acid hydrolysis ([(M + H)^+^] *m/z* = 171.0, 77%, Scheme 9).

**Scheme 10:**
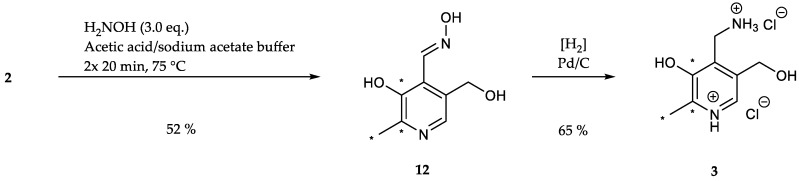
Preparation of [^13^C_3_]-PM (**3**) was accomplished starting from [^13^C_3_]-PL (**2**) in two steps.

For the preparation of [^13^C_3_]-PM, the aldehyde **2** was firstly transformed into the respective oxime **12** using hydroxylamine hydrochloride ([(M + H)^+^] *m/z* = 186.1, 52%, Scheme 10).

Reportedly, an excess addition of sodium acetate (NaAc) is necessary to ensure a positive reaction outcome (65%, Table 6, entry 1) [33,46,73]. The reduction of the amount of sodium acetate towards stoichiometric quantities resulted in no conversion of the starting material, thus confirming the results from literature (Table 6, entry 2).

Because of the presumable pH-dependence of the reaction, the next step in the optimization involved the substitution of NaAc with a sodium acetate/acetic acid buffer (pH = 4.8), which instantly led to a higher yield (71%, Table 6, entry 3). Variation of the reaction time showed that a slightly longer reaction time (2 × 20 min) improved the reaction outcome (79%), whereas further prolongation (2 × 30 min) had no further enhancing effect (78%, Table 6, entry 4/5). Moreover, a second addition of buffer appeared to be necessary for the continuance of the reaction (61%, Table 6, entry 6). Reduction of the amount of hydroxylamine hydrochloride resulted in a decrease of yield (45%, Table 6, entry 7). Finally, the [^13^C_3_]-Oxime **12** was hydrogenated using H_2_ and Pd/C to obtain [^13^C_3_]-PM (**3**, [(M + H)^+^] *m/z* = 172.1, 65%, Scheme 10).

## 3. Materials and Methods

### 3.1. General Information

Reactions sensitive to air or moisture were carried out in dried glassware under a positive pressure of argon using standard Schlenk techniques. Solvents were distilled and stored over molecular sieves prior to use. Chemicals received from commercial sources (Acros, Sigma-Aldrich and Fluka from Darmstadt, Germany) were used without further purification unless stated otherwise. [^13^C_3_]-propionic acid was purchased from Sigma-Aldrich and used without further purification.

### 3.2. Column Chromatography/TLC

Column chromatography was performed on silica gel 60 (Merck, 230–240 mesh) with the eluent mixtures given for the corresponding procedures. Thin-layer chromatography (TLC) was performed using silica-coated aluminum plates (silica gel 60). The substances were detected by UV (λ = 254 nm, 366 nm) or after visualization with CAM (cerium ammonium molybdate; 0.5 g Ce(NH_4_)_2_(NO_3_)_6_ and 24 g of (NH_4_)_6_Mo_7_O_24_∙4H_2_O in 28 mL H_2_SO_4_ stirred for 1 h) or KMnO_4_ (1.5 g KMnO_4_, 10 g K_2_CO_3_ and 1.25 mL 10% NaOH in 200 mL H_2_O) solution.

### 3.3. NMR

NMR spectra were recorded either on a Bruker AV III system (400 MHz, Bruker, Rheinstetten, Germany) or on a Bruker AV III system (500 MHz, Bruker, Rheinstetten, Germany). ^1^H- and ^13^C-NMR spectra were recorded at 400 or 500 MHz and at 101 or 126 MHz, respectively. ^1^H- and ^13^C-NMR spectroscopic chemical shifts δ are reported in parts per million (ppm) relative to residual proton signal. All coupling constants (*J*) are reported in Hertz (Hz). The following abbreviations or combinations thereof were used to explain multiplicities: s = singlet, d = doublet, t = triplet, q = quartet, m = multiplet. For all detailed data see Supplementary Materials.

### 3.4. LC-MS/MS

LC-MS/MS was carried out on a Shimadzu LC-30A Prominence system (Shimadzu, Kyoto, Japan) with the mobile phase combinations water/acetonitrile or water/methanol. The injection volume was 1 µL. The LC was interfaced with a triple quadrupole ion trap mass spectrometer (LCMS-8050, Shimadzu). Data acquisition was performed with LabSolutions software 5.80 (Shimadzu). 

### 3.5. Experimental Procedures

*Ethyl 2-bromo[^13^C_3_]propionate* (**5**): Thionyl chloride (4.63 g, 38.9 mmol, 2.82 mL, 3.00 eq.) and [^13^C_3_]propionic acid (1.00 g, 12.9 mmol, 1.00 eq.) were heated to reflux for 2.5 h. Bromine (1.56 g, 19.5 mmol, 0.50 mL, 1.50 eq.) was added over the course of 2.5 h and the heating continued overnight. After cooling to 0 °C, ethanol (1.49 g, 32.4 mmol, 1.90 mL, 2.50 eq.) was added during 30 min and stirring continued for 24 h at rt. 60 mL brine were added at 0 °C and the resulting suspension extracted with diethyl ether (3 × 50 mL). The collected organic phases were washed successively with sat. NaHCO_3_ (1 × 50 mL), sat. Na_2_S_2_O_3_ (1 × 50 mL) and brine (1 × 50 mL). After drying over Na_2_SO_4_ and evaporation of the solvent, the crude product was distilled under reduced pressure resulting in a colorless clear oil or directly used in the next reaction.^1^H NMR (400 MHz, CDCl_3_, 292 K): *δ* [ppm] = 4.36 (m, *J* = 154.9, 7.0 Hz, 1 H), 4.23 (qdd, *J* = 7.2, 3.3, 1.9 Hz, 2 H), 1.82 (ddt, *J* = 130.6, 6.9, 4.7 Hz, 3 H), 1.30 (m, 3 H); ^13^C NMR (101 MHz, CDCl_3_, 292 K): *δ* [ppm] = 170.4 (^13^C, d, *J* = 64.2 Hz), 62.1 (s), 40.4 (^13^C, dd, *J* = 64.2, 36.3 Hz), 21.8 (^13^C, d, *J* = 36.3 Hz), 14.0 (s). The obtained data of the unlabeled substances matched those reported in [74].

*Potassium phthalimide*: Phthalimide (2.00 g, 13.6 mmol, 1.00 eq.) was heated to reflux in ethanol (50 mL) and poured into a solution of potassium hydroxide (0.76 g, 13.6 mmol, 1.00 eq.) in 0.75 mL water and 2.30 ml ethanol. The resulting suspension was cooled to 0 °C and filtered *via Buchner* funnel. After washing the greenish residuum with ethanol (1 × 25 mL) and aceton (2 × 25 mL), the remaining solvent was evaporated under reduced pressure to obtain the product as greenish crystals (1.96 g, 10.6 mmol, 71%). ^1^H NMR (400 MHz, D_2_O, 292 K): *δ* [ppm] = 7.58–7.31 (m, 4 H).

*Ethyl 2-(1,3-dioxoisoindolin-2-yl)[^13^C_3_]propionate***(6)**: Potassium phthalimide (4.99 g, 26.9 mmol, 2.50 eq.) was added to a solution of ethyl 2-bromo)[^13^C_3_]propionate (1.98 g, 10.7 mmol, 1.00 eq.) in 150 mL acetonitrile and heated to reflux for 24 h. The solvent was removed under reduced pressure and the residue diluted with dichloromethane (75 mL) and water (75 mL). After separation of the phases, the aqueous phase was extracted with dichloromethane (2 × 50 mL). The combined organic phases were washed with water (2 × 40 mL) and brine (1 × 30 mL), dried over Na_2_SO_4_ and the solvent evaporated under reduced pressure to obtain the crude product (2.28 mg, 9.11 mmol) as a white solid, which was directly used in the next reaction. ^1^H-NMR (400 MHz, CDCl_3_, 292 K): *δ* [ppm] = 7.87 (m, 2 H), 7.74 (m, 2 H), 5.00 (m, *J* = 136.6, 7.5, 5.4 Hz, 1 H), 4.21 (qdd, *J* = 7.1, 3.3, 2.5 Hz, 2 H), 1.69 (ddt, *J* = 130.5, 7.3, 4.6 Hz, 3 H), 1.23 (t, *J* = 7.1 Hz, 3 H); ^13^C NMR (101 MHz, CDCl_3_, 292 K): *δ* [ppm] = 169.8 (^13^C, d, *J* = 61.9 Hz), 167.5 (s), 134.2 (s), 132.1 (s), 123.6 (s), 62.0 (s), 47.7 (^13^C, dd, *J* = 61.8, 37.6 Hz), 15.4 (^13^C, d, *J* = 37.5 Hz), 14.2 (s); ESI-MS calcd: [(M + H)^+^] *m/z* 251.2, [(M + Na)^+^] *m/z* 273.2, found: [(M + H)^+^] *m/z* 251.3, [(M + Na)^+^] *m/z* 273.0.

*[^13^C_3_]Alanine hydrochloride* (**7**): Acetic acid (25 mL) and 6 N HCl (130 mL) were added to ethyl 2-(1,3-dioxoisoindolin-2-yl)[^13^C_3_]propionate (2.28 mg, 9.11 mmol, 1.00 eq.) and heated to reflux overnight. The solvent was evaporated under reduced pressure and the residue dissolved in water. The aqueous phase was washed with ethyl acetate (3 × 25 mL) and the solvent evaporated under reduced pressure to obtain the product as a white solid (1.33 g, 10.4 mmol, 78%). ^1^H NMR (400 MHz, D_2_O, 292 K): *δ* [ppm] = 4.10 (ddq, *J* = 146.3, 13.0, 6.9 Hz, 1 H), 1.56 (ddt, *J* = 130.9, 7.3, 4.5 Hz, 3 H); ^13^C NMR (101 MHz, D_2_O, 292 K): *δ* [ppm] = 172.9 (^13^C, dd, *J* = 58.9, 1.5 Hz), 48.8 (^13^C, dd, *J* = 59.0, 34.3 Hz), 15.3 (^13^C, d, *J* = 34.0 Hz); ESI-MS calcd: [(M − HCl + H)^+^] *m/z* 93.1,found: [(M − HCl + H)^+^] *m/z* 93.0.

*Butyl [^13^C_3_]alaninate hydrochloride***(8)**: Thionyl chloride (1.85 g, 15.5 mmol, 1.13 mL, 1.50 eq.) was added to a solution of [^13^C_3_]alanine hydrochloride (1.33 g, 10.4 mmol, 1.00 eq.) in *n*-butanol (100 mL) at 0 °C and heated to reflux for 2 h. The solvent was evaporated under reduced pressure and the residue crystallized from diethyl ether/pentane to receive the product as an off-white solid (1.61 g, 8.70 mmol, 83%). ^1^H NMR (400 MHz, CDCl_3_, 292 K): *δ* [ppm] = 8.74 (br, 3 H), 4.22 (m, *J* = 146.2, 6.6 Hz, 1 H), 4.19 (m, 2 H), 1.72 (m, *J* = 131.1, 7.0, 4.6 Hz, 3 H), 1.64 (dq, *J* = 8.7, 6.7 Hz, 2 H), 1.38 (h, *J* = 7.4 Hz, 2 H), 0.93 (t, *J* = 7.4 Hz, 3 H); ^13^C NMR (101 MHz, CDCl_3_, 292 K): *δ* [ppm] = 170.1 (^13^C, dd, *J* = 62.0, 1.5 Hz), 66.4 (s), 49.4 (^13^C, dd, *J* = 62.1, 34.1 Hz), 30.5 (s), 19.1 (s), 16.3 (^13^C, d, *J* = 35.0 Hz), 13.8 (s); ESI-MS calcd: [(M – HCl + H)^+^] *m/z* 149.1,found: [(M − HCl + H)^+^] *m/z* 149.0. The obtained data of the unlabeled substances matched those reported in [62].

*Butyl-N-formyl-[^13^C_3_]alaninate* (**9**): A mixture of butyl [^13^C_3_]alaninate hydrochloride (1.61 g, 8.70 mmol, 1.00 eq.) and trimethyl orthoformiate (4.62 g, 4.76 mL, 43.5 mmol, 5.00 eq.) was heated to 165 °C for 2 h. The volatile compounds were removed under reduced pressure to obtain the product as a yellow oil (1.24 g, 7.06 mmol, 84%).^1^H-NMR (400 MHz, CDCl_3_, 292 K): *δ* [ppm] = 8.19 (d, *J* = 5.1 Hz, 1 H), 6.20 (s, 1 H), 4.67 (ddt, *J* = 143.1, 12.4, 7.1 Hz, 1 H), 4.17 (tt, *J* = 6.6, 2.8 Hz, 2 H), 1.70–1.56 (m, 2 H + 1.5 H), 1.39 (dq, *J* = 14.6, 7.3 Hz, 2 H), 1.28 (dt, *J* = 7.1, 4.5 Hz, 1.5 H), 0.94 (t, *J* = 7.4 Hz, 3 H); ^13^C-NMR (101 MHz, CDCl_3_, 292 K): *δ* [ppm] = 172.8 (^13^C, dd, *J* = 61.2, 1.2 Hz), 160.4 (s), 65.7 (s), 47.06 (^13^C, dd, *J* = 61.3, 34.8 Hz), 30.6 (s), 19.1 (s), 18.8 (^13^C, dd, *J* = 34.9, 1.3 Hz), 13.8 (s); ESI-MS: calcd: [(M + H)^+^] *m/z* 177.2, found: [(M + H)^+^] *m/z* 177.2.The obtained data of the unlabeled substances matched those reported in [43].

*5-Butoxy-4-[^13^C_1_]methyl[4,5-^13^C_2_]oxazole* (**10**): A solution of butyl-*N*-formyl-[^13^C_3_]alaninate (1.24 g, 7.06 mmol, 1.00 eq.) in dichloromethane was added to a homogenous mixture of celite (2 g), calcium oxide (2 g) and P_2_O_5_ (2.5 g, 17.6 mmol, 2.5 eq.) in dichloromethane (100 mL) under inert atmosphere. The mixture was stirred at room temperature for 30 min followed by heating to reflux for 48 h. After the first 24 h, additional 2.5 eq. P_2_O_5_ were added at rt and the heating was continued. A sat. Aqueous solution of NaHCO_3_ was added at 0 °C, the mixture was filtered and the aqueous phase extracted with dichloromethane (3 × 50 mL). The combined organic phases were washed with brine (1 × 25 mL), dried over Na_2_SO_4_ and the solvent removed under reduced pressure. The crude product was purified *via* column chromatography (pentane/diethyl ether = 3/1) to obtain the product (0.68 g, 4.33 mmol, 62%) as yellow oil. ^1^H-NMR (400 MHz, CDCl_3_, 292 K): *δ* [ppm] = 7.37 (dd, *J* = 6.9, 4.4 Hz, 1 H), 4.08 (td, *J* = 6.6, 2.9 Hz, 2 H), 2.04 (ddd, *J* = 128, 7.2, 4.6 Hz, 3 H), 1.76–1.64 (m, 2 H), 1.52–1.41 (m, 2 H), 0.96 (t, *J* = 7.4 Hz, 3 H); ^13^C-NMR (101 MHz, CDCl_3_, 292 K): *δ* [ppm] = 154.6 (^13^C, dd, *J* = 97.5, 7.2 Hz), 142.4–142.0 (m), 112.0 (^13^C, dd, *J* = 97.5, 56.4 Hz), 74.4–74.2 (m), 31.5 (d, *J* = 2.4 Hz), 18.9 (s), 13.8 (s), 10.1 (^13^C, dd, *J* = 56.4, 7.2 Hz); ESI-MS calcd: [(M + H)^+^] *m/z* 159.1,found: [(M + H)^+^] *m/z* 159.1; TLC: *R*_f_ = 0.34 (pentane/diethyl ether = 3/1 [KMnO_4_]).The obtained data of the unlabeled substances matched those reported in [42].

*2-[^13^C_1_]Methyl-3-hydroxy-4,5-epoxydimethyl[2,3-^13^C_2_]pyridine* (**11**): 5-Butoxy-4-[^13^C_1_]methyl[4,5-^13^C_2_]oxazole (0.68 g, 4.33 mmol, 1.00 eq.), 2,5-dihydrofuran (9.63 mL, 130 mmol, 30.0 eq.) and trichloroacetic acid (0.15 g, 0.95 mmol) were heated to 210 °C for 5 h in a pressure vial and thereafter allowed to cool to room temperature overnight. The reaction mixture was concentrated under reduced pressure and the crude product purified *via* column chromatography (diethyl ether/methanol = 10/0.5) to obtain the product (0.40 mg, 2.63 mmol, 61%) as brownish solid. ^1^H-NMR (400 MHz, MeOD, 292 K): *δ* [ppm] = 7.78 (d, *J* = 9.0 Hz, 1 H), 5.06 (d, *J* = 11.0 Hz, 4 H), 2.42 (ddd, *J* = 127.8, 6.5, 2.8 Hz, 3 H); ^13^C-NMR (101 MHz, MeOD, 292 K): *δ* [ppm] = 149.57 (^13^C, d, *J* = 66.6 Hz), 145.99 (^13^C, dd, *J* = 66.8, 50.6 Hz), 137.16 (s), 137.08 (s), 130.4–130.1 (m), 72.85 (d, *J* = 2.4 Hz), 72.33 (m), 17.64 (^13^C, dd, *J* = 50.6, 4.4 Hz); ESI-MS calcd: [(M + H)^+^] *m/z* 155.1, [(M − H)^−^] *m/z* 153.0,found: [(M + H)^+^] *m/z* 155.1, [(M − H)^−^] *m/z* 153.0; TLC: *R*_f_ = 0.27 (diethyl ether/methanol = 10/0.5 [KMnO_4_]).

*[^13^C_3_]Pyridoxine (PN, 1)*: A solution of 2-[^13^C_1_]methyl-3-hydroxy-4,5-epoxydimethyl[2,3-^13^C_2_]pyridine (0.40 mg, 2.63 mmol, 1.00 eq.) in 6 mL 48% HBr_aq._ was heated to reflux for one hour. After cooling to rt, the solvent was evaporated under reduced pressure. Water (25 mL) and freshly prepared AgCl (8 g) were added and the reaction heated to reflux for one hour. After filtration over a Celite-Pad and washing with water, the solvent was removed under reduced pressure and the crude product purified *via* column chromatography (dichloromethane/methanol = 10/1) to obtain an off-white powder (0.45 g, 2.15 mmol, 82%). ^1^H-NMR (400 MHz, D_2_O, 292 K): *δ* [ppm] = 8.16 (d, *J* = 7.0 Hz, 1 H), 5.00 (d, *J* = 3.9 Hz, 2 H), 4.80 (s, 2 H), 2.64 (ddd, *J* = 131.4, 6.8, 3.1 Hz, 3 H); ^13^C-NMR: (101 MHz, D_2_O, 292 K): *δ* [ppm] = 150.5 (^13^C, dd, *J* = 72.7, 1.5 Hz), 140.5 (^13^C, dd, *J* = 72.9, 46.4 Hz), 138.5, 134.5, 127.4, 55.8 (d, *J* = 3.5 Hz), 54.5 (d), 12.1 (^13^C, dd, *J* = 46.5, 1.6 Hz); ESI-MS calcd: [(M − HCl + H)^+^] *m/z* 173.1, [(M − HCl − H)^−^] *m/z* 171.1,found: [(M − HCl + H)^+^] *m/z* 173.0, [(M – HCl − H)^−^] *m/z* 171.5; TLC: *R*_f_ = 0.28 (dichloromethane/methanol = 10/1 [KMnO_4_]).

*[^13^C_3_]N-(Pyridoxylidene)methylamine* (**13**): A solution of potassium permanganate (0.18 g, 1.16 mmol, 0.60 eq.) in distilled water (20 mL) was added portion wise to a solution of [^13^C_3_]pyridoxine (0.40 g, 1.93 mmol, 1.00 eq.) in distilled water (10 mL) over the course of 1 h followed by heating to 70 °C for 1 h. Afterwards, the reaction mixture was reduced to 1/3 of its volume under reduced pressure. Methylamine hydrochloride (0.78 g, 11.5 mmol, 6.00 eq.) was added to the suspension and the pH adjusted to 8. The resulting mixture was heated to 70 °C for 30 min and afterwards stirred at room temperature for another 48 hours. The aqueous phase was extracted with dichloromethane (3 × 50 mL). The collected organic phases were dried over Na_2_SO_4_ and the solvent evaporated under reduced pressure to obtain the product as yellow-brownish crystals (0.12 g, 0.66 mmol, 34%). ^1^H-NMR (400 MHz, CDCl_3_, 292 K): *δ* [ppm] = 8.86 (dt, *J* = 5.6, 1.6 Hz, 1 H), 7.83 (d, *J* = 10.6 Hz, 1 H), 4.78 (s, 2 H), 3.57 (d, *J* = 1.5 Hz, 3 H), 2.51 (ddd, *J* = 127.9, 6.6, 2.7 Hz, 3 H); ^13^C-NMR (101 MHz, CDCl_3_, 292 K): *δ* [ppm] = 164.0 (s), 155.4 (^13^C, dd, *J* = 65.0, 4.8 Hz), 151.1 (^13^C, dd, *J* = 65.0, 51.6 Hz), 137.7 (s), 131.01 (s), 121.1 (s), 60.8 (d, *J* = 2.0 Hz), 46.3 (s), 19.1 (^13^C, dd, *J* = 51.6, 4.7 Hz); ESI-MS calcd: [(M + H)^+^] *m/z* 184.1, [(M − H)^−^] *m/z* 182.2,found: [(M + H)^+^] *m/z* 184.0, [(M − H)^−^] *m/z* 182.5.

*[^13^C_3_]Pyridoxal (PL, 2)*: [^13^C_3_]*N*-(pyridoxylidene)methylamine (0.11 g, 0.64 mmol, 1.00 eq.) was dissolved in 10 mL 1 N HCl and stirred for 1 h at rt. The crude product was purified *via* column chromatography (dichloromethane/methanol = 8/1) to obtain ^13^C_3_-PL as white solid (0.08 g, 0.49 mmol, 77%). ^1^H-NMR (400 MHz, D_2_O, 292 K): *δ* [ppm] = 8.19 (d, *J* = 6.5 Hz, 1 H), 6.77 (s, 1 H), 5.36 (d, *J* = 13.9 Hz, 1 H), 5.22 (d, *J* = 13.9 Hz, 1 H), 2.68 (ddd, *J* = 131.6, 6.7, 3.2 Hz, 3 H); ^13^C-NMR (101 MHz, D_2_O, 292 K): *δ* [ppm] = 149.0 (^13^C, dd, *J* = 73.1, 1.6 Hz), 143.9 (^13^C, dd, *J* = 73.1, 46.0 Hz), 14.1 (^13^C, dd, *J* = 46.0, 4.8 Hz); ESI-MS: calcd: [(M+H)^+^] *m/z* 171.1, [(M−H)^−^] *m/z* 169.1,found: [(M+H)^+^] *m/z* 171.0, [(M−H)^−^] *m/z* 169.4; TLC: *R*_f_ = 0.3 (dichloromethane/methanol = 8/1 [KMnO_4_]).

*[^13^C_3_]N-(Pyridoxylidene)hydroxylamine* (**12**): Hydroxylamine hydrochloride (0.09 g, 1.30 mmol, 3.00 eq.) was added to a solution of [^13^C_3_]pyridoxal (74.9 mg, 0.44 mmol, 1.00 eq.) in 5 mL sodium acetate/acetic acid buffer (pH = 4.8). After heating the reaction mixture for 20 min at 75 °C, 1 mL buffer was added followed by a further heating period of 20 min at 75 °C. The crude product was purified *via* column chromatography (dichloromethane/methanol = 10/1) to obtain the oxime as white solid (42.6 mg, 0.23 mmol, 52%). ^1^H-NMR (400 MHz, MeOD, 292 K): *δ* [ppm] = 8.64 (dd, *J* = 5.8, 1.2 Hz, 1 H), 7.89 (dd, *J* = 10.7, 1.5 Hz, 1 H), 4.69 (s, 2 H), 2.45 (ddd, *J* = 127.9, 6.6, 2.8 Hz, 3 H); ^13^C-NMR (101 MHz, MeOD, 292 K): *δ* [ppm] = 152.4 (^13^C, dd, *J* = 67.9, 4.5 Hz), 148.9 (^13^C, dd, *J* = 67.9, 51.0 Hz), 139.3 (s), 133.6 (s), 122.5 (s), 60.4 (d, *J* = 2.5 Hz), 18.4 (^13^C, dd, *J* = 51.0, 4.4 Hz); ESI-MS calcd: [(M+H)^+^] *m/z* 186.1, [(M−H)^−^] *m/z* 184.5,found: [(M+H)^+^] *m/z* 186.1, [(M−H)^−^] *m/z* 184.1; TLC: *R*_f_ = 0.27 (dichloromethane/methanol = 10/1 [KMnO_4_]).

*[^13^C_3_]Pyridoxamine (PM, 3)*: Palladium/coal (24.8 mg, 30% wt, 0.07 mmol, 0.60 eq.) was added to a solution of [^13^C_3_]*N*-(pyridoxylidene)hydroxylamine (21.4 mg, 0.11 mmol, 1.00 eq.) in 4 mL methanol. The reaction vessel was flushed with H_2_ until complete conversion of the starting material (indicated through TLC). 0.5 mL 4 N HCl were added and the mixture filtered. After evaporation of the solvent, the crude product was crystallized from methanol/diethyl ether to obtain [^13^C_3_]-PM dihydrochloride as white solid (18.2 mg, 0.07 mmol, 65%). ^1^H-NMR (400 MHz, D_2_O, 292 K): *δ* [ppm] = 8.24 (d, *J* = 7.0 Hz, 1 H), 4.87 (s, 2 H), 4.44 (d, *J* = 4.3 Hz, 2 H), 2.72 (ddd, *J* = 131.5, 6.5, 3.3 Hz, 3 H); ^13^C-NMR (101 MHz, D_2_O, 292 K): *δ* [ppm] = 153.8 (^13^C, d, *J* = 69.9 Hz), 142.9 (^13^C, dd, *J* = 70.1, 46.3 Hz), 58.6 (d, *J* = 3.2 Hz), 34.6 (s), 15.0 (^13^C, dd, *J* = 46.5, 1.9 Hz); ESI-MS calcd: [(M+H)^+^] *m/z* 172.1, [(M−H)^−^] *m/z* 170.1,found: [(M+H)^+^] *m/z* 172.1, [(M−H)^−^] *m/z* 170.2.

## 4. Conclusions

In summary, the first synthetic route for the preparation of [^13^C_3_]-labelled vitamers of the B6 group (PN, PL, PM) starting from [^13^C_3_]-propionic acid was presented. Hereby, higher alkyl homologues showed a positive impact on the yield of various intermediates: Next to switching from TEO towards TMO, and thus facilitating the purification, increasing the alkyl chain of the alcohol resulted in nearly quantitative conversions towards the formylated alanine ester. Furthermore, we investigated the influence, on the one hand, of CaO as an additive in comparison to MgO, which represents the commonly used reagent in this type of reaction, and, on the other hand, higher alkyl residues—in this regard the first time with *n*-BuOH under the noted conditions—on the formation of 5-Alkoxy-4-methyl oxazoles, both factors leading to a significant increase of the reaction outcome. While utilization of hydroxylamine and sodium acetate belongs to the catalogue of common craftsmanship regarding the preparation of PN-oxime, we came across the enhancing effect on the yield using a sodium-acetate buffer instead of neat NaAc. Finally, choosing this route reduces the cost for the preparation of labelled product due to fully labelled propionic acid being a cheaper commercially available substrate than alanine, which is mostly used in the literature.

## Figures and Tables

**Table 1:** Reaction conditions for the preparation of the α-brominated propionic ester [51].

Entry	Alc R ^c^	t_SOCl2_ [h]	t_Br2_ [h]	t_Alc_ [h]	eq. Br_2_	eq. Alc	PA [g]	Yield [%] ^b^
1	Ethyl	2.5	3.5	12	1.05	2.5	5	76
2	Ethyl	2.5	5.5	12	1.05	2.5	5	88
3	Ethyl	2.5	2d	24	1.50	2.5	5	87
4	Ethyl	2.5	5.5	12	1.50	2.5	2	76
5	Ethyl	4.0	18	24	1.50	11	2	71
6	Ethyl	2.5	5.5	12 + 1 ^a^	1.50	13	1	36
7	Ethyl	2.5	17	5d	1.50	2.5	1	73
8	Methyl	2.5	24	24	1.20	5.0	1	67
9	*t*-Butyl	2.5	5.5	12	1.05	2.5	1	/
10	*n*-Butyl	2.5	5.5	12	1.05	1.6	5	69

^a^ Reflux for 1 h. ^b^ Isolated yield. ^c^ Alkyl of respective ester.

**Table 2:** Reaction conditions for the N-formylation of alanine alkyl esters.

Entry	Alc R	TMO [eq.]	T [°C]	t [h]	Yield [%]
1	Ethyl	3.00	150	2	96
2	Ethyl	4.10	160	1.5	89
3	Ethyl	5.00	160	2	97
4	Ethyl	6.90	160	1.5	76
5	Ethyl	7.00	160	2	92
6	*n*-Propyl	3.80	160	1.5	87
7	*n*-Propyl	4.00	160	1.5	87
8	*n*-Propyl	5.00	165	1.5	91
9	*n*-Propyl	5.00	150	2	96
10	*n*-Propyl	6.30	160	2	95
11	*n*-Butyl	3.00	150	2	99
12	*n*-Butyl	3.00	160	2	99
13	*n*-Butyl	4.00	160	1.5	97
14	*n*-Butyl	5.00	150	2	96
15	*n*-Butyl	5.00	160	2	99
16 ^a^	*n*-Butyl	5.00	165	2	84

^a^ Reaction performed with [^13^C_3_]-labelled starting material.

**Table 3:** Reaction conditions for the cyclisation of 5-alkoxy-4-methyl-oxazoles.

Entry	Alc R	P_2_O_5_ [eq.] ^b^	Additive [eq.]	Yield [%]
1 ^a^	Ethyl	5.0	MgO, 4.8	22
2	Ethyl	2.5 + 2.5 (24 h)	MgO, 5.4	27
3	Ethyl	2.5 + 2.5 (24 h)	MgO, 10.7	40
4	Ethyl	2.5 + 2.5 (24 h)	SnCl_2_, 1.1	19
5	Ethyl	2.5 + 2.5 (24 h)	CaO, 4.0	46
6	Ethyl	2.5 + 2.5 (24 h)	CaO, 4.2	48
7	*n*-Propyl	2.5 + 2.5 (24 h)	CaO, 3.7	54
8	*n*-Butyl	2.5 + 2.5 (24 h)	CaO, 4.9	70
9 ^c^	*n*-Butyl	2.5 + 2.5 (24 h)	CaO, 5.0	62

^a^ Reaction performed without celite. ^b^ Second addition after 24 h. ^c^ Reaction performed with [^13^C_3_]-labelled starting material.

**Table 4:** Reaction conditions for the Diels-Alder reaction.

Entry	Alc R	2,5-DHF [eq.]	CCl_3_COOH [eq.]	t [h]	T [°C]	Yield [%]
1	Ethyl	30	1%	5	200	57
2	*n*-Propyl	20	-	5	190	27
3	*n*-Butyl	40	1%	6	210	72
4 ^a^	*n*-Butyl	30	1%	6	210	61

^a^ Reaction performed with [^13^C_3_]-labelled starting material.

**Table 5:** Reaction conditions for the oxidation/imination of PN.

Entry	Oxidant [eq.]	T [°C] ^a^	t [min] ^a^	CH_3_NH_2_ [eq.]	T [°C] ^b^	t [min] ^b^	t [d] at rt	Yield [%]
1	MnO_2_ [31]	70	60	6	70	30	2	34
2	MnO_2_ 9.6	70	60	6	70	30	2	42
3	MnO_2_ 9.6	70	20	6	70	30	1	22
4	KMnO_4_ 0.4	rt	60	6	80	10	10	35
5	KMnO_4_ 0.8	rt	60	6	80	30	8	23
6	KMnO_4_ 0.5	70	60	6	75	35	2	24
7	KMnO_4_ 0.7	70	60	10	75	30	2	11
8	KMnO_4_ 0.8	70	60	6	65	30	3	21
9	KMnO_4_ 1.0	70	60	6	60	30	2	18
10	KMnO_4_ 1.4	60	60	3	70	30	2	13
11	KMnO_4_ 1.6	70	60	6	70	2 h 15 min	3	34
12	KMnO_4_ 0.8	40	24 h	6	70	30	2	19
13	KMnO_4_ 0.5	70	45	6	70	30	2	42
14	KMnO_4_ 0.5	70	20	6	70	30	1	41
15 ^c^	KMnO_4_ 0.6	70	60	6	70	30	2	34

^a^ Referring to the oxidation step. ^b^ Referring to the imination step. ^c^ Reaction performed with [^13^C_3_]-labelled starting material.

**Table 6:** Reaction conditions for the oxime preparation.

Entry	t [min]	Hydroxylamine [eq.]	Yield [%]
1	2 × 10 ^a^	3.0	65
2	2 × 10 ^a^	3.0	0
3	2 × 10 ^b^	3.0	71
4	2 × 20 ^b^	3.0	79
5	2 × 30 ^b^	3.0	78
6	1 × 40 ^b^	3.0	61
7	2 × 10 ^b^	1.5	45
8 ^c^	2 × 20 ^b^	3.0	52

^a^ NaAc (11 eq. for entry 1 *resp.* 1 eq. entry 2) used. ^b^ NaAc/Acetic acid buffer used. ^c^ Reaction performed with [^13^C_3_]-labelled starting material.

## Supplementary Materials

Experimental procedures and NMR-spectra of the new substances are available online. Figure S1. ^1^H spectrum of ethyl 2-bromo)(^13^C_3_)propionate. Figure S2. ^13^C spectrum of ethyl 2-bromo)(^13^C_3_)propionate. Figure S3. ^1^H spectrum of ethyl 2-(1,3-dioxoisoindolin-2-yl)(^13^C_3_)propionate. Figure S4. ^13^C spectrum of ethyl 2-(1,3-dioxoisoindolin-2-yl)(^13^C_3_)propionate. Figure S5. ^1^H spectrum of (^13^C_3_)alanine hydrochloride. Figure S6. ^13^C spectrum of (^13^C_3_)alanine hydrochloride. Figure S7. ^1^H spectrum of butyl (^13^C_3_)alaninate hydrochloride. Figure S8. ^13^C spectrum of butyl (^13^C_3_)alaninate hydrochloride. Figure S9. ^1^H spectrum of butyl-N-formyl-(^13^C_3_)alaninate. Figure S10. ^13^C spectrum of butyl-N-formyl-(^13^C_3_)alaninate. Figure S11. ^1^H spectrum of 5-butoxy-4-(^13^C_1_)methyl(4,5-^13^C_2_)oxazole. Figure S12. ^13^C spectrum of 5-butoxy-4-(^13^C_1_)methyl(4,5-^13^C_2_)oxazole. Figure S13. ^1^H spectrum of 2-(^13^C_1_)methyl-3-hydroxy-4,5-epoxydimethyl(2,3-^13^C_2_)pyridine. Figure S14. ^13^C spectrum of 2-(^13^C_1_)methyl-3-hydroxy-4,5-epoxydimethyl(2,3-^13^C_2_)pyridine. Figure S15. ^1^H spectrum of (^13^C_3_)pyridoxine. Figure S16. ^13^C spectrum of (^13^C_3_)pyridoxine. Figure S17. ^1^H spectrum of (^13^C_3_)N-(pyridoxylidene)methylamine. Figure S18. ^13^C spectrum of (^13^C_3_)N-(pyridoxylidene)methylamine. Figure S19. ^1^H spectrum of (^13^C_3_)pyridoxal. Figure S20. ^13^C spectrum of (^13^C_3_)pyridoxal. Figure S21. ^1^H spectrum of (^13^C_3_)N-(pyridoxylidene)hydroxylamine. Figure S22. ^13^C spectrum of (^13^C_3_)N-(pyridoxylidene)hydroxylamine. Figure S23. ^1^H spectrum of (^13^C_3_)pyridoxamine. Figure S24.^13^C spectrum of (^13^C_3_)pyridoxamine.

## Abbreviations:

2,5-DHF	2,5-Dihydrofuran
CCl_3_COOH	Trichloroacetic acid
IBX	2-Iodoxybenzoic acid
P_2_O_5_	Phosphorus pentoxide
PA	Propionic acid
PN	Pyridoxine
PL	Pyridoxal
PM	Pyridoxamine
PLP	Pyridoxal phosphate
PMP	Pyridoxamine phosphate
NMR	Nuclear magnetic resonance
TEMPO	2,2,6,6-Tetramethylpiperidinyloxyl
