# Supplementary material for: Synthesis of [13C3]-B6 Vitamers Labelled at Three Consecutive Positions Starting from [13C3]-Propionic Acid
Source: Molecules. 2018 Aug 23;23(9):2117. doi: 10.3390/molecules23092117 (PMC6225105; doi:10.3390/molecules23092117)
Supplement: Supplementary file 1 [file molecules-23-02117-s001.pdf]

Synthesis of $^{13}C_3$-B6 vitamers labelled at three consecutive positions starting from $^{13}C_3$-propionic acid

Electronic Supplementary Information

Thomas Bachmann and Michael Rychlik*

Lehrstuhl für Analytische Lebensmittelchemie, Technische Universität München,
Maximus-von-Imhof-Forum 2, 85354 Freising, Germany

NMR spectra of new compounds

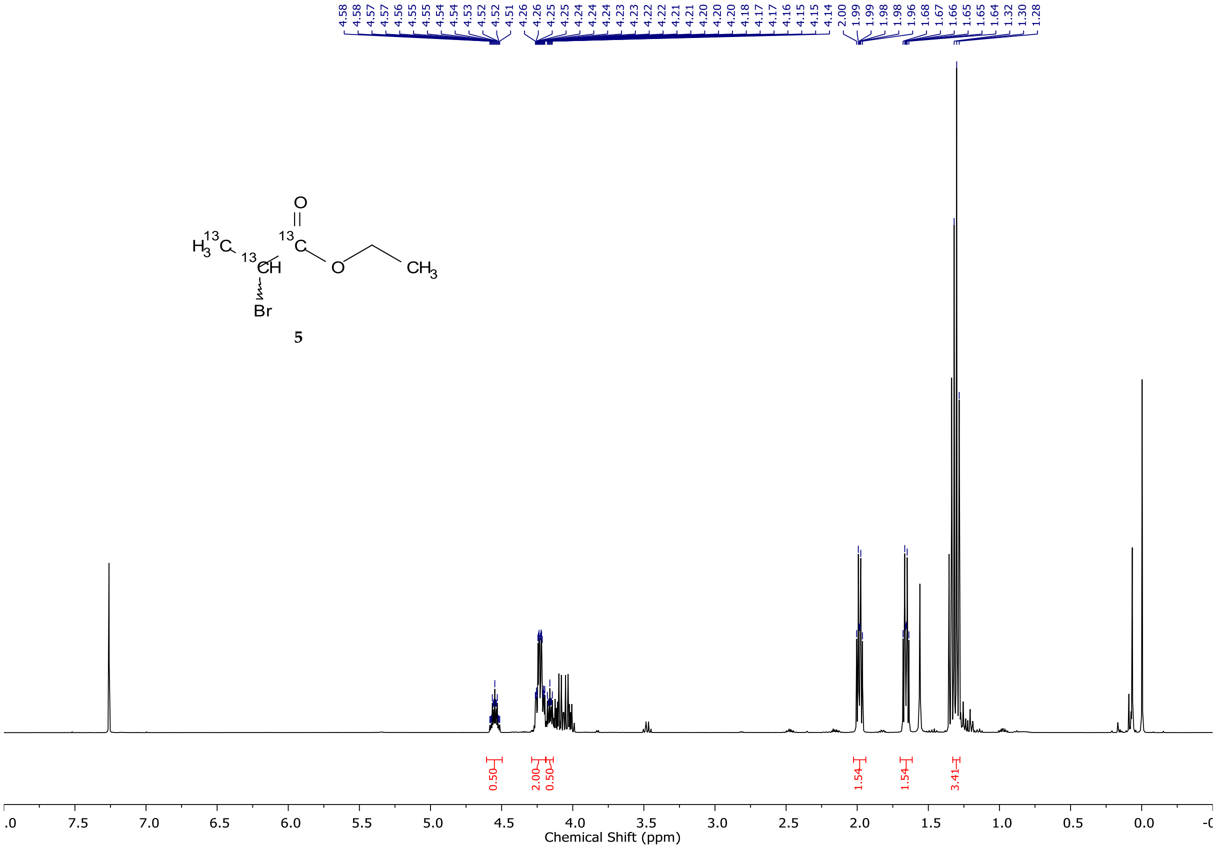

S1. ^{1}H spectrum of ethyl 2-bromo)($^{13}C_3$)propionate

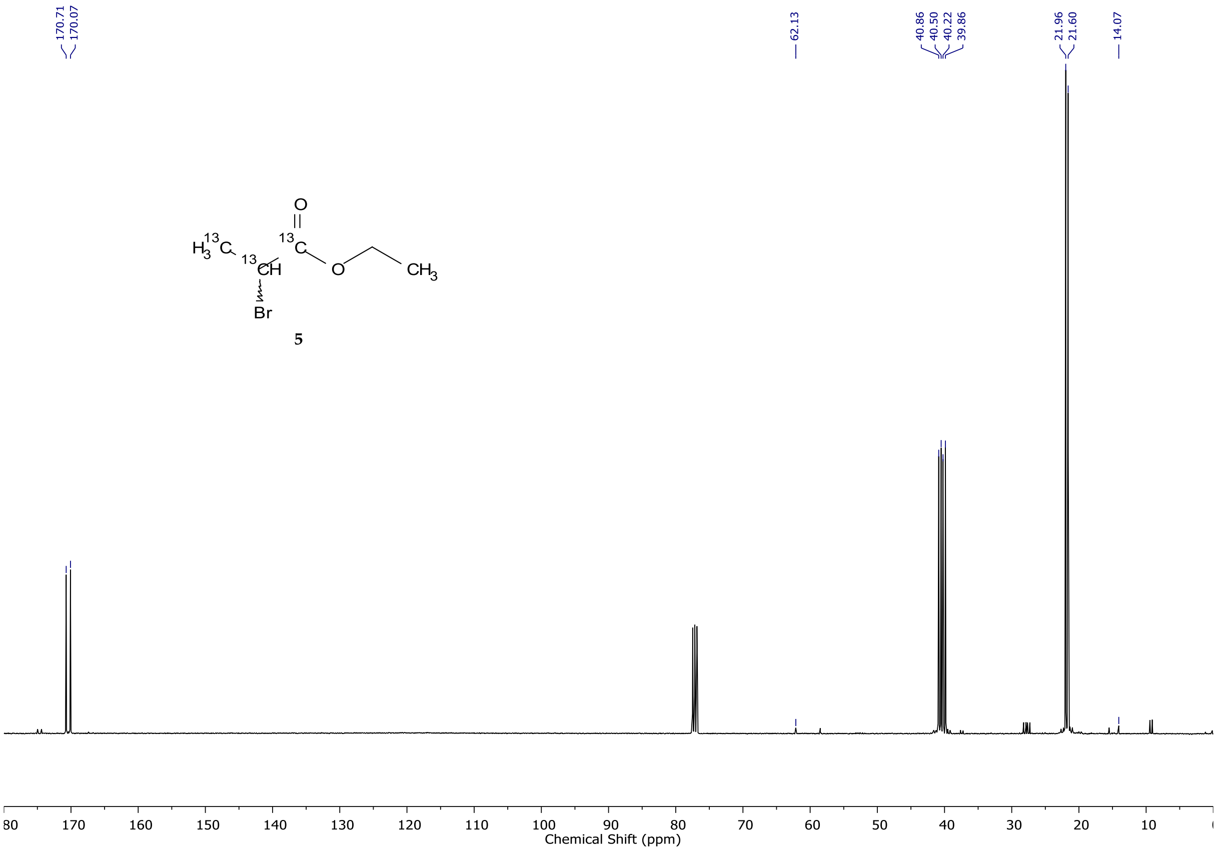

S2. ^{13}C spectrum of ethyl 2-bromo)($^{13}C_3$)propionate

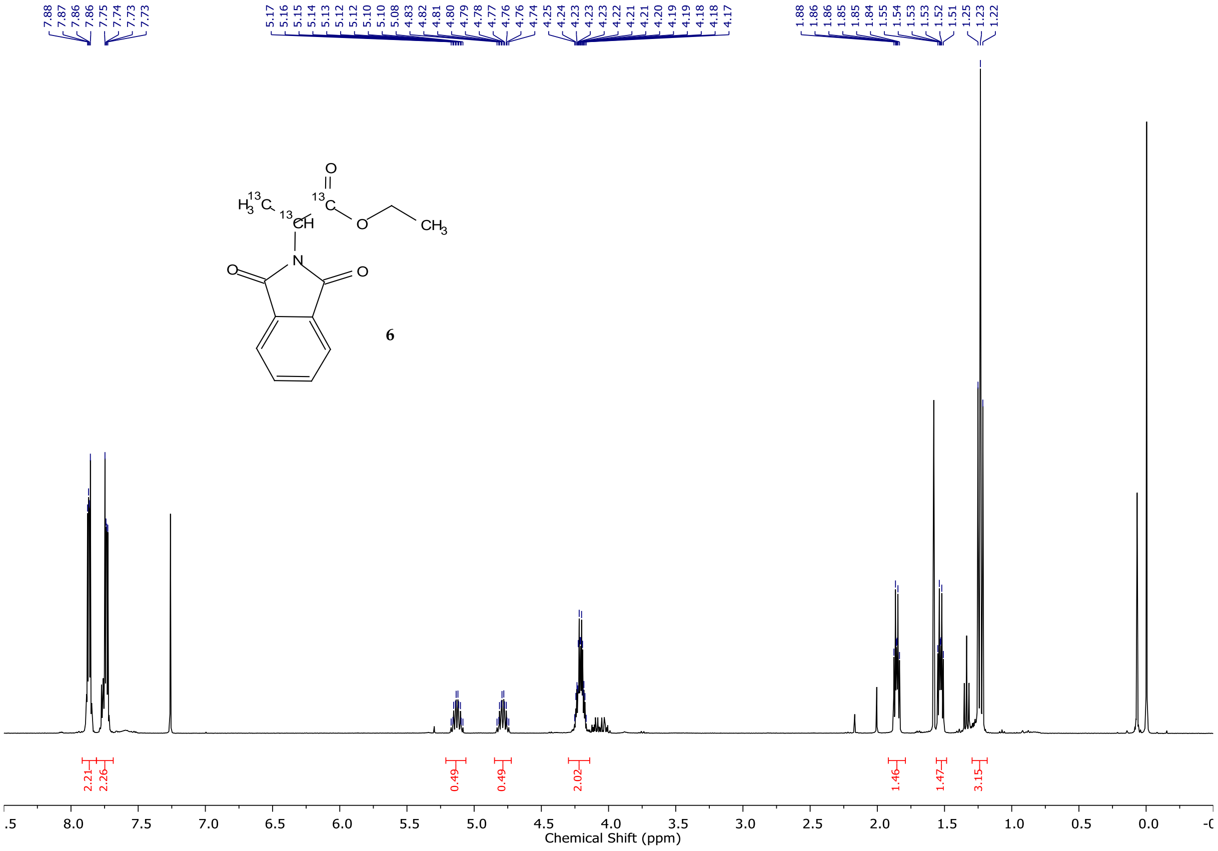

S3. ^{1}H spectrum of ethyl 2-(1,3-dioxoisoindolin-2-yl)($^{13}C_3$)propionate

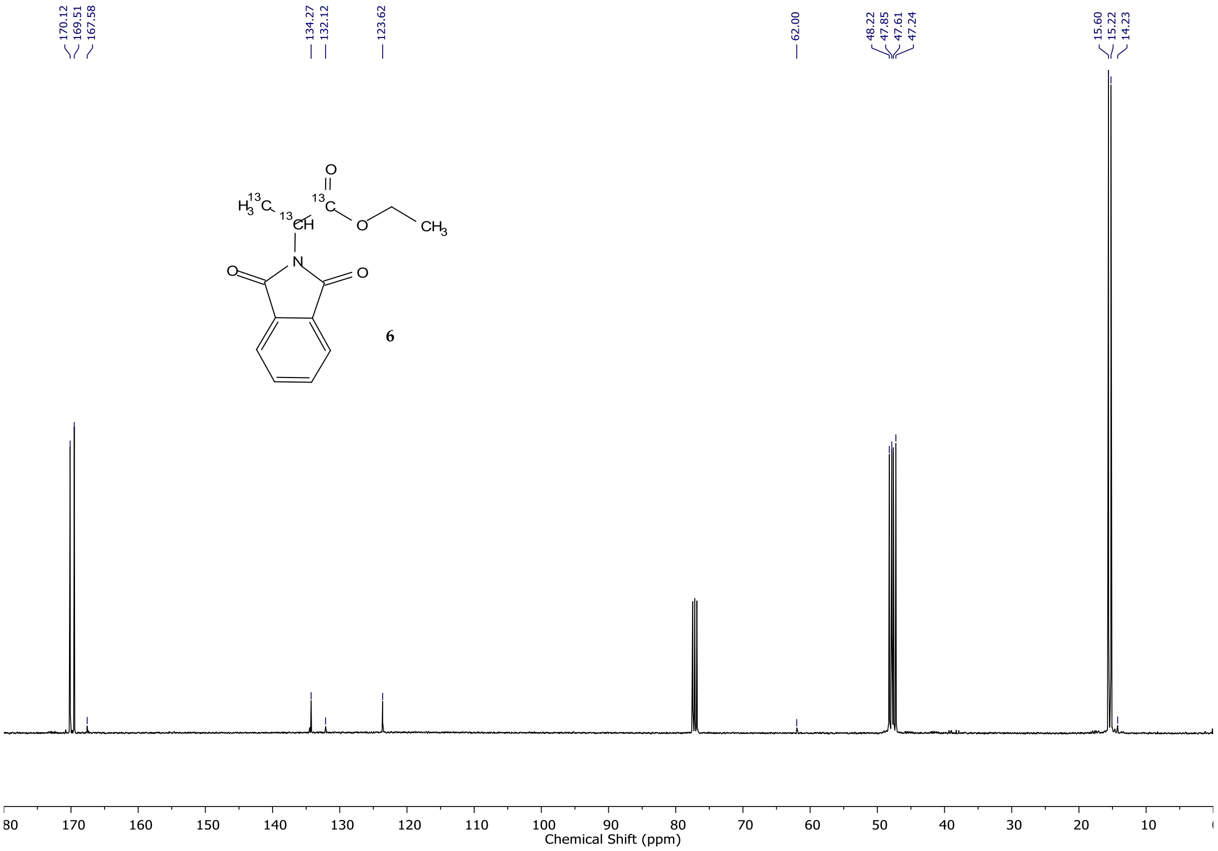

S4. ^{13}C spectrum of ethyl 2-(1,3-dioxoisoindolin-2-yl)($^{13}C_3$)propionate

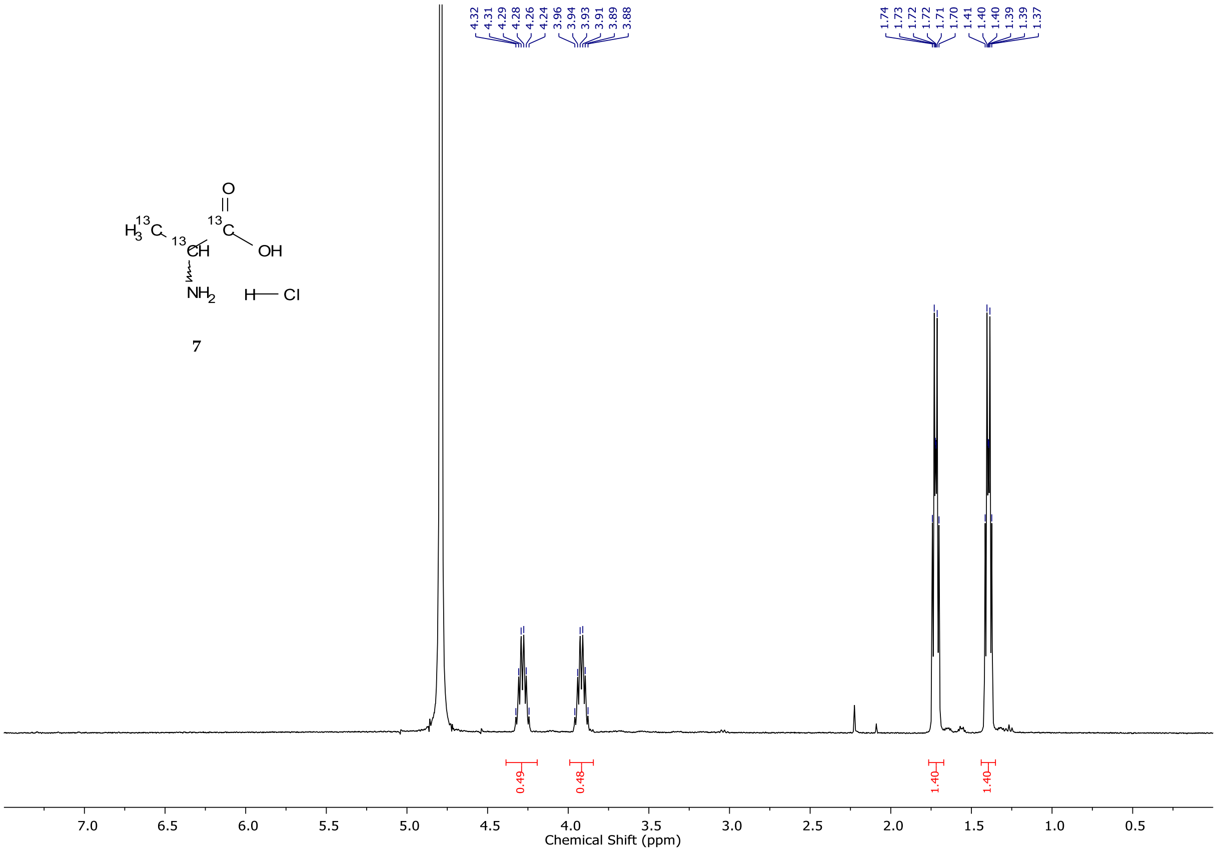

S5. ^{1}H spectrum of ($^{13}C_3$)alanine hydrochloride

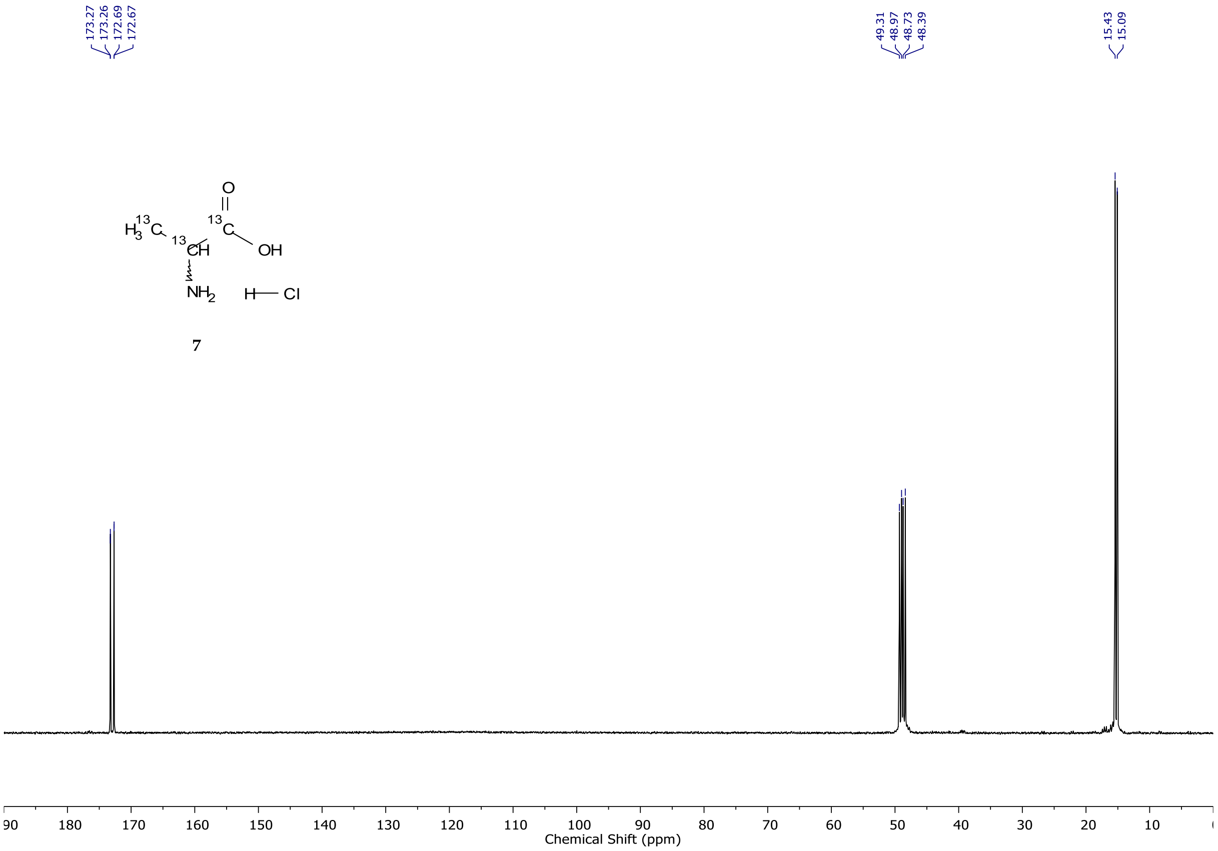

S6. ^{13}C spectrum of ($^{13}C_3$)alanine hydrochloride

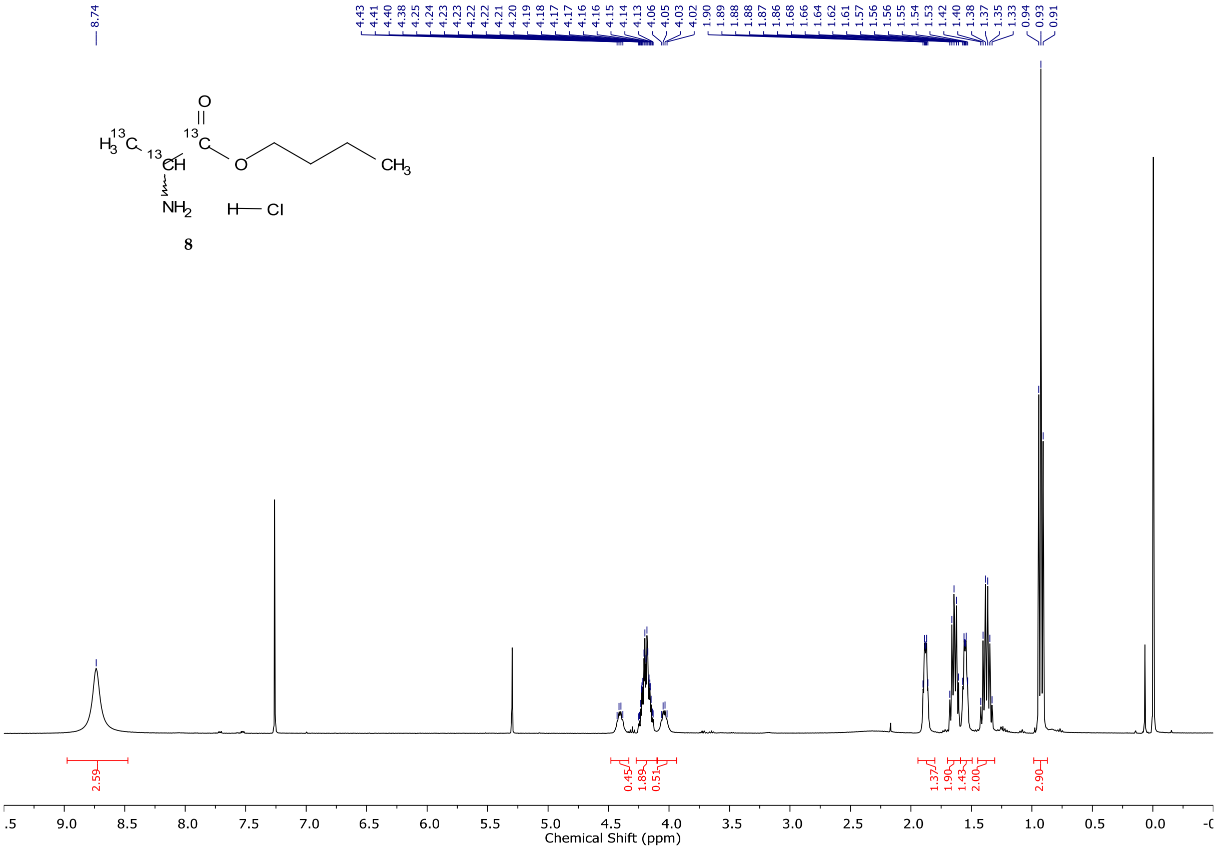

S7. ^{1}H spectrum of butyl ($^{13}C_3$)alaninate hydrochloride

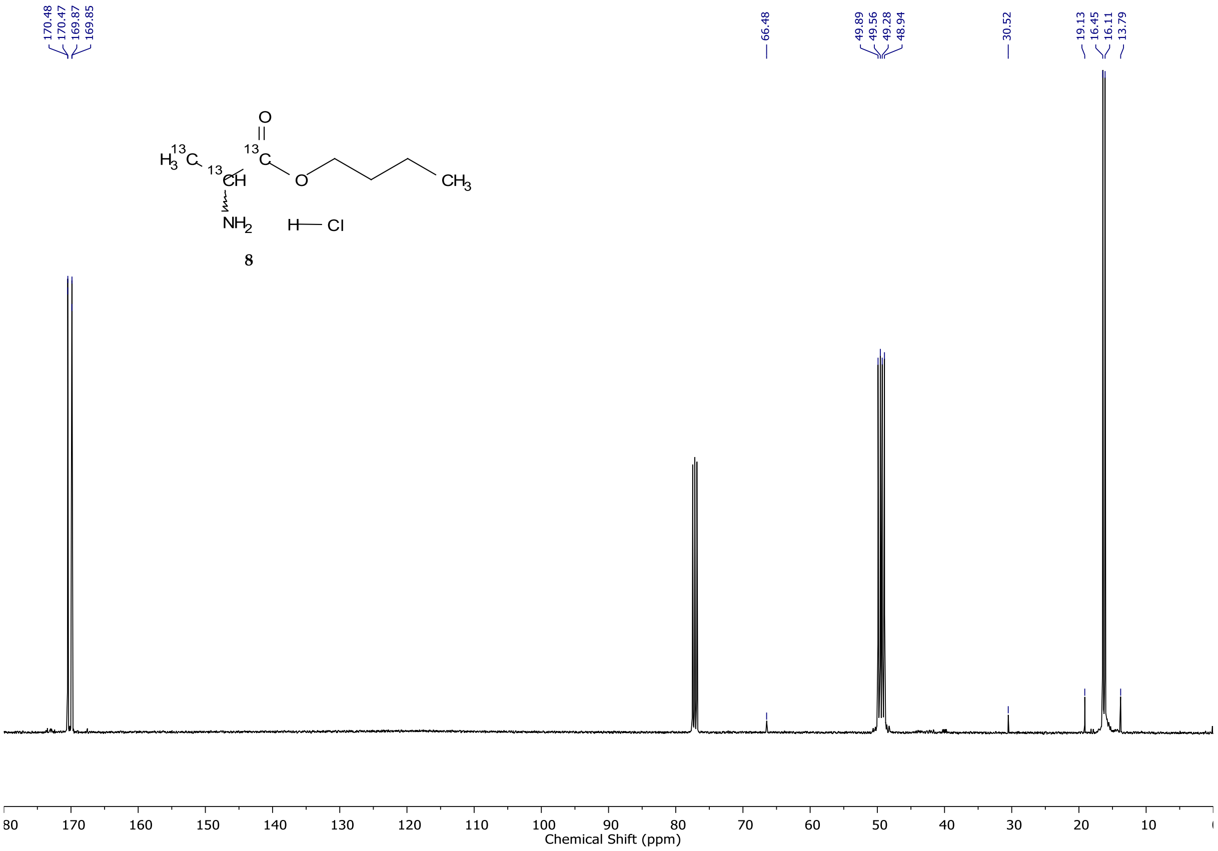

S8. ^{13}C spectrum of butyl ($^{13}C_3$)alaninate hydrochloride

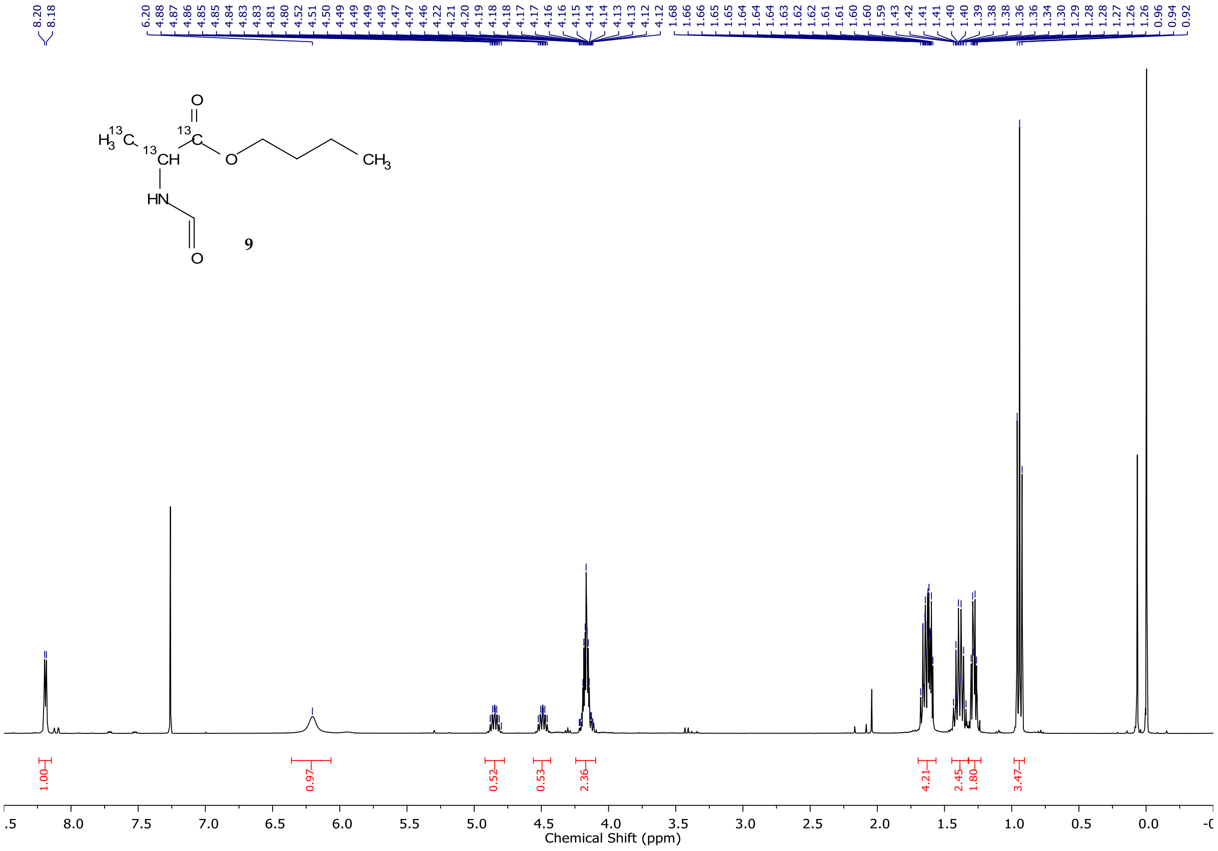

S9. ^{1}H spectrum of butyl-*N*-formyl-($^{13}C_3$)alaninate

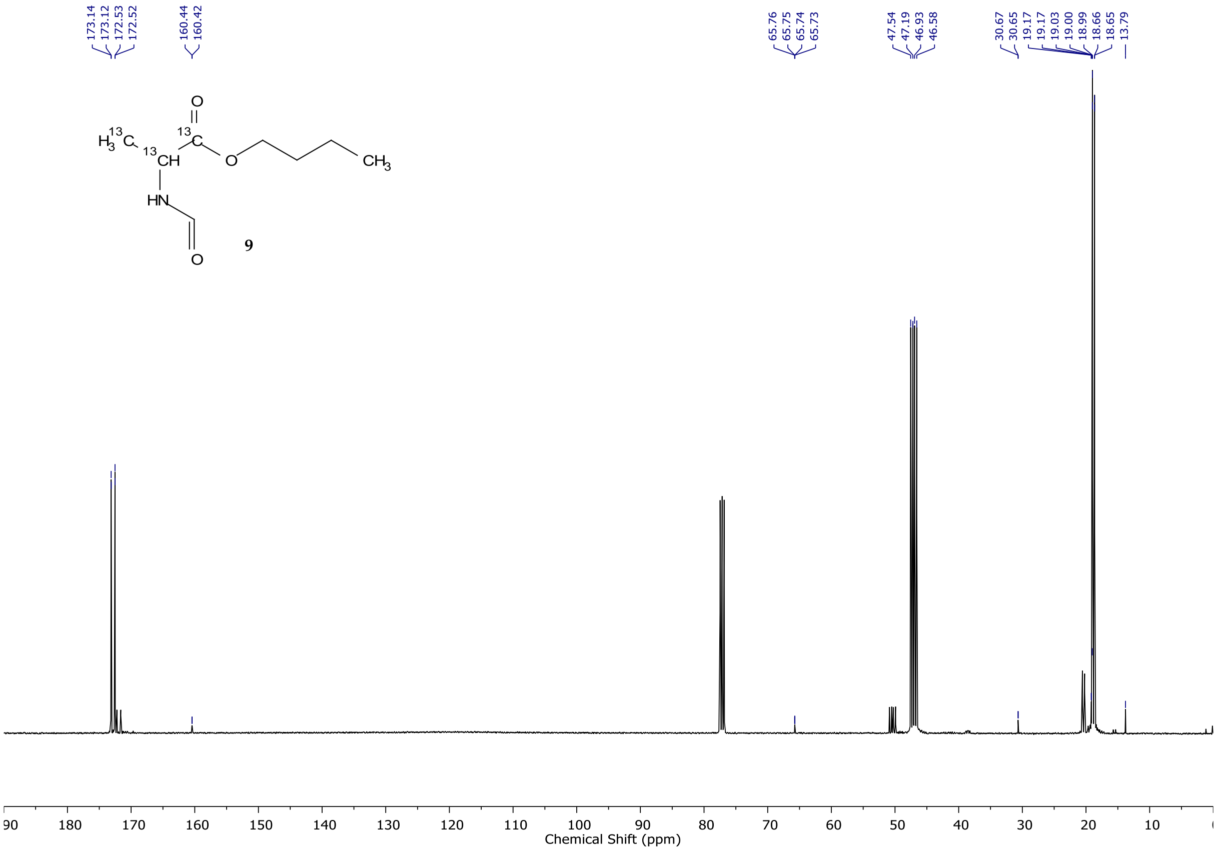

S10. ^{13}C spectrum of butyl-*N*-formyl-($^{13}C_3$)alaninate

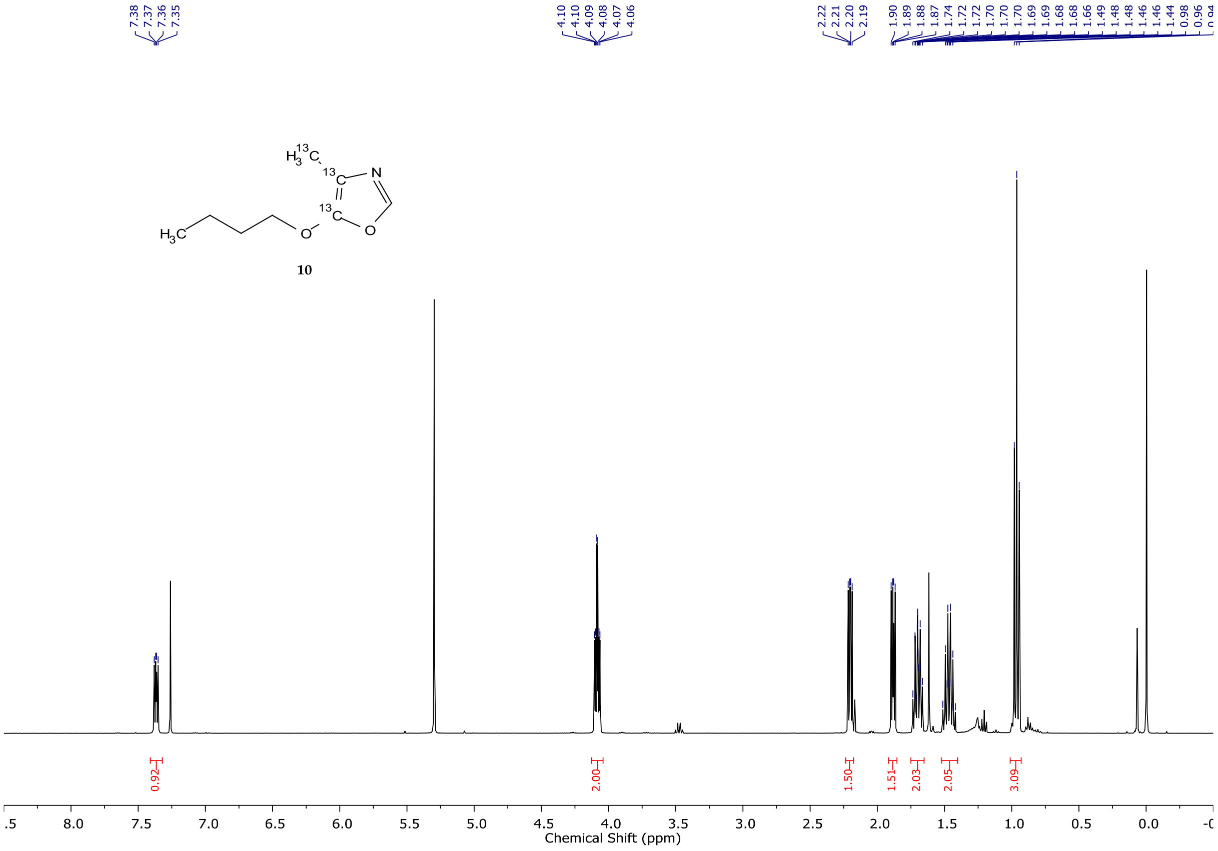

S11. ^{1}H spectrum of 5-butoxy-4-($^{13}C_1$)methyl(4,5-$^{13}C_2$)oxazole

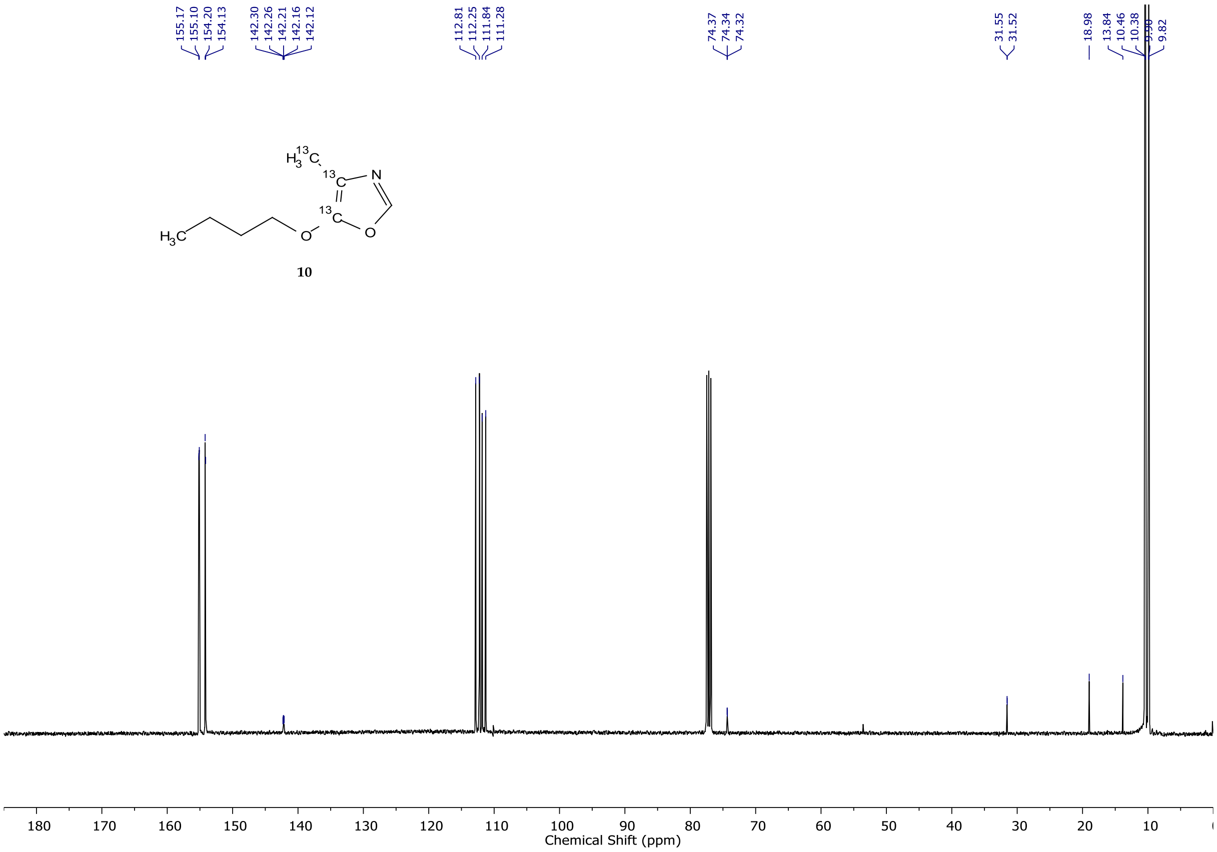

S12. ^{13}C spectrum of 5-butoxy-4-($^{13}C_1$)methyl(4,5-$^{13}C_2$)oxazole

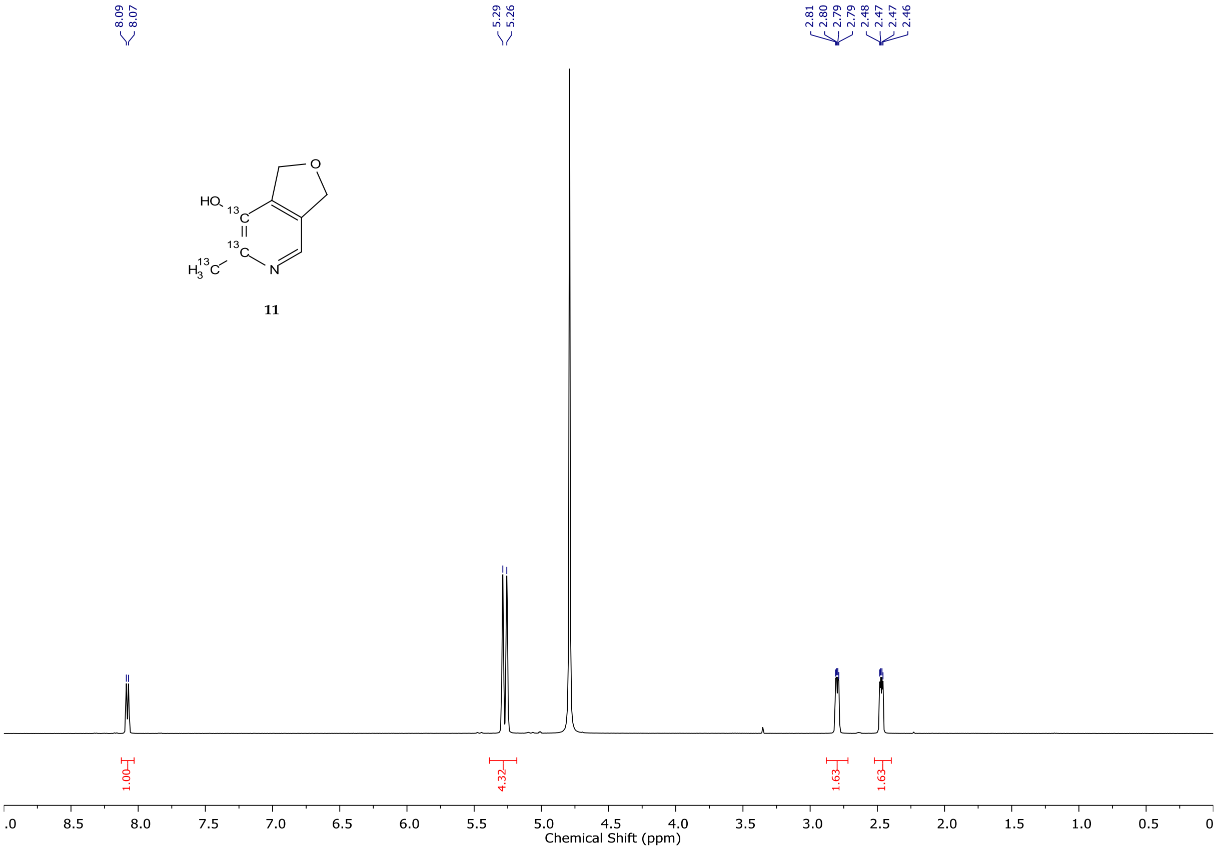

S13. ^{1}H spectrum of 2-($^{13}C_1$)methyl-3-hydroxy-4,5-epoxydimethyl(2,3-$^{13}C_2$)pyridine

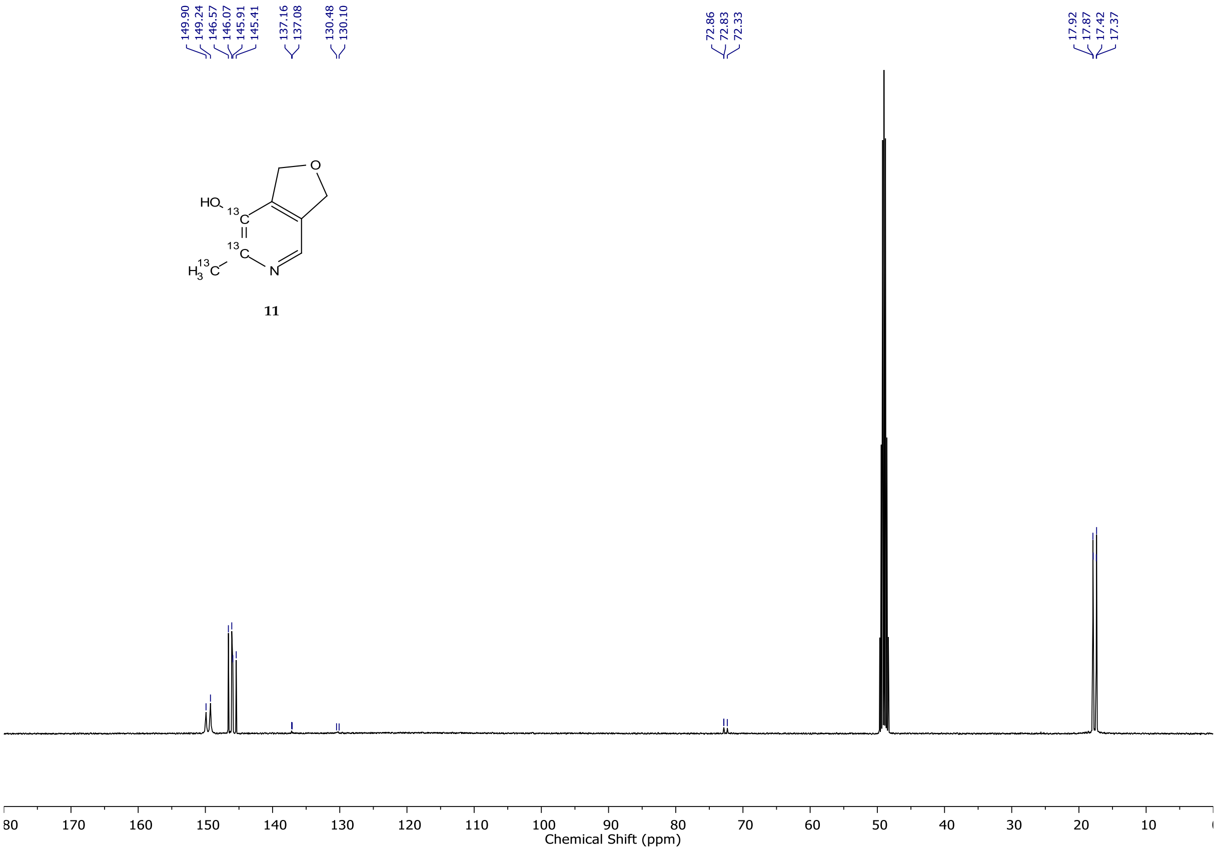

S14. ^{13}C spectrum of 2-($^{13}C_1$)methyl-3-hydroxy-4,5-epoxydimethyl(2,3-$^{13}C_2$)pyridine

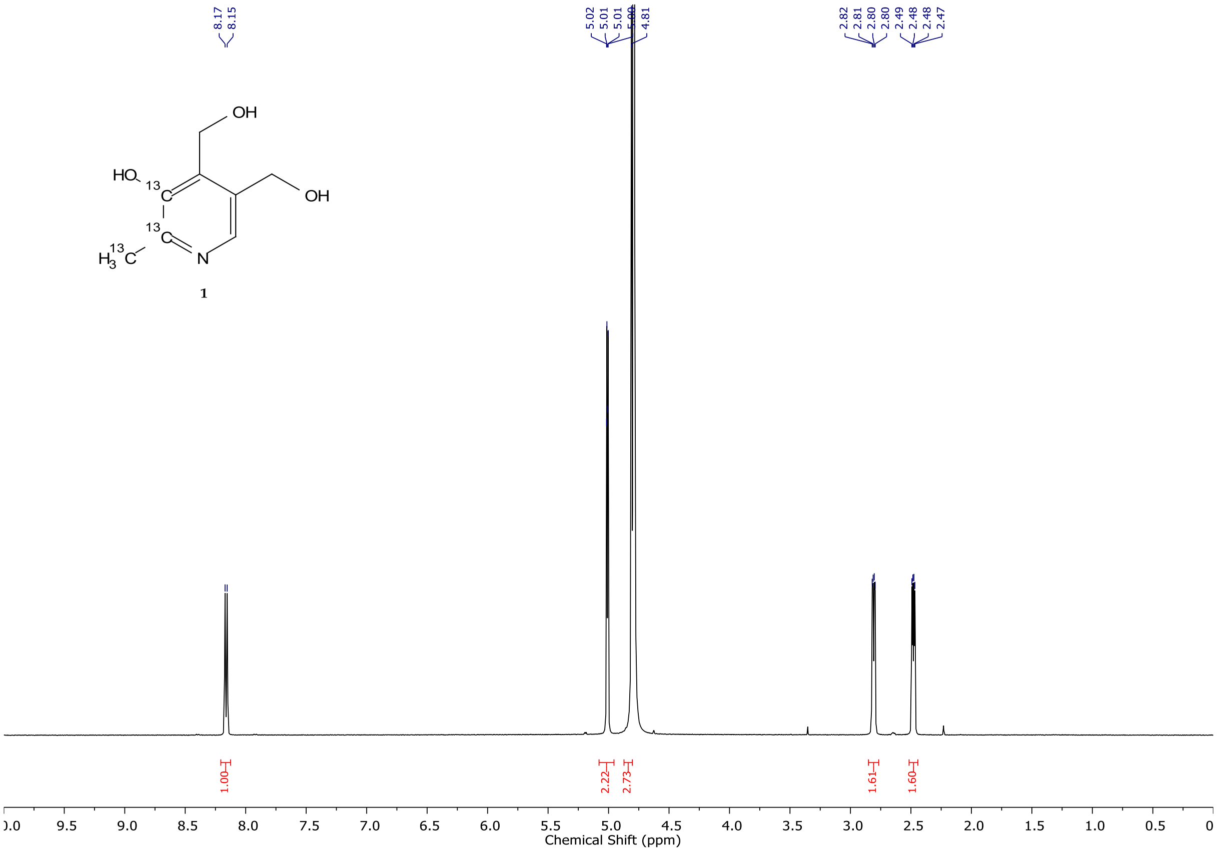

S15. ^{1}H spectrum of ($^{13}C_3$)pyridoxine

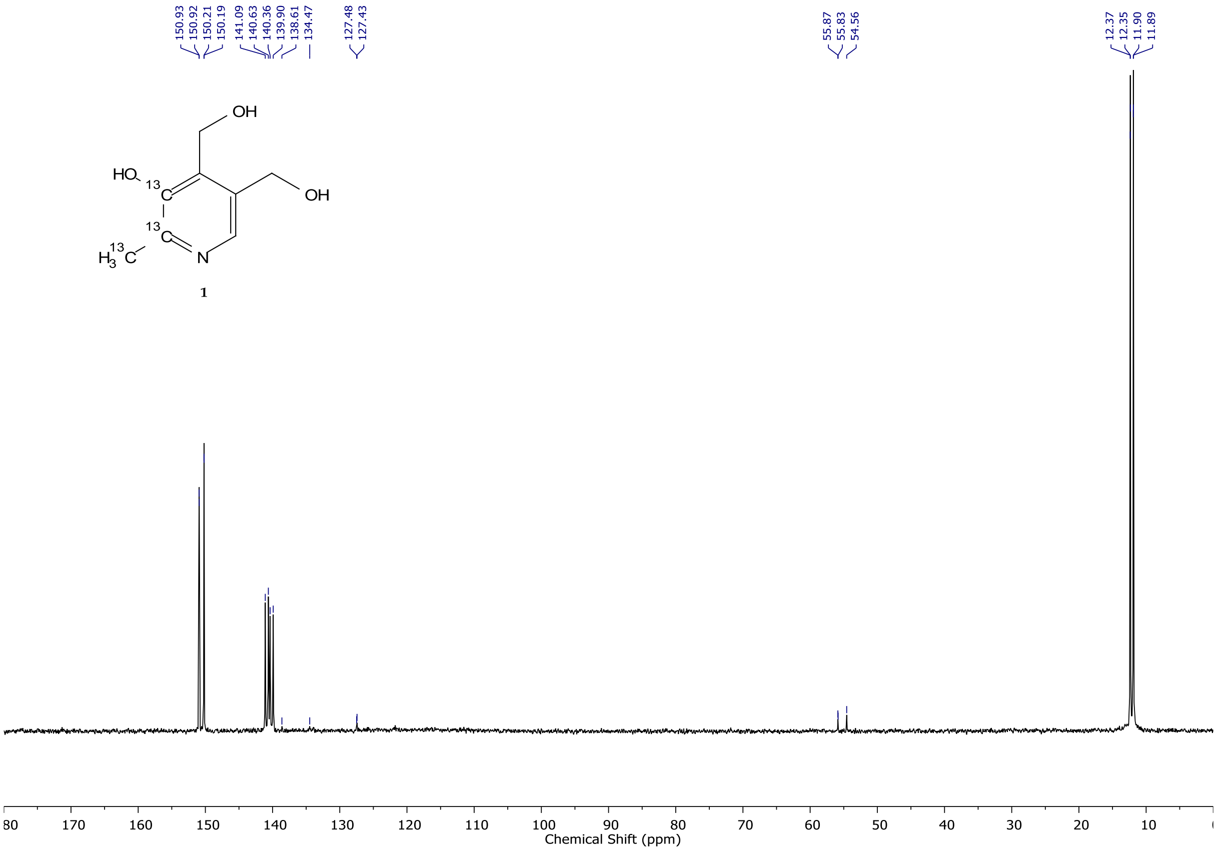

S16. ^{13}C spectrum of ($^{13}C_3$)pyridoxine

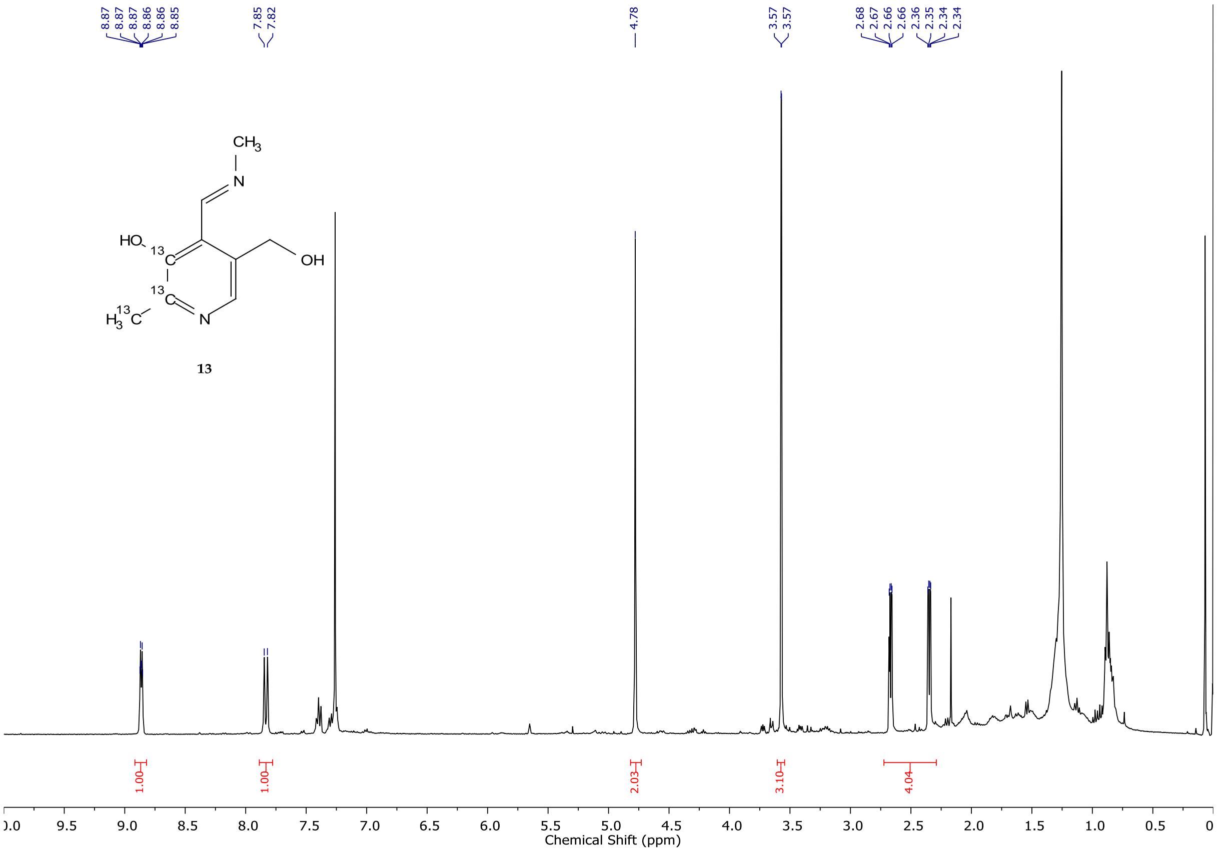

S17. ^{1}H spectrum of ($^{13}C_3$)*N*-(pyridoxylidene)methylamine

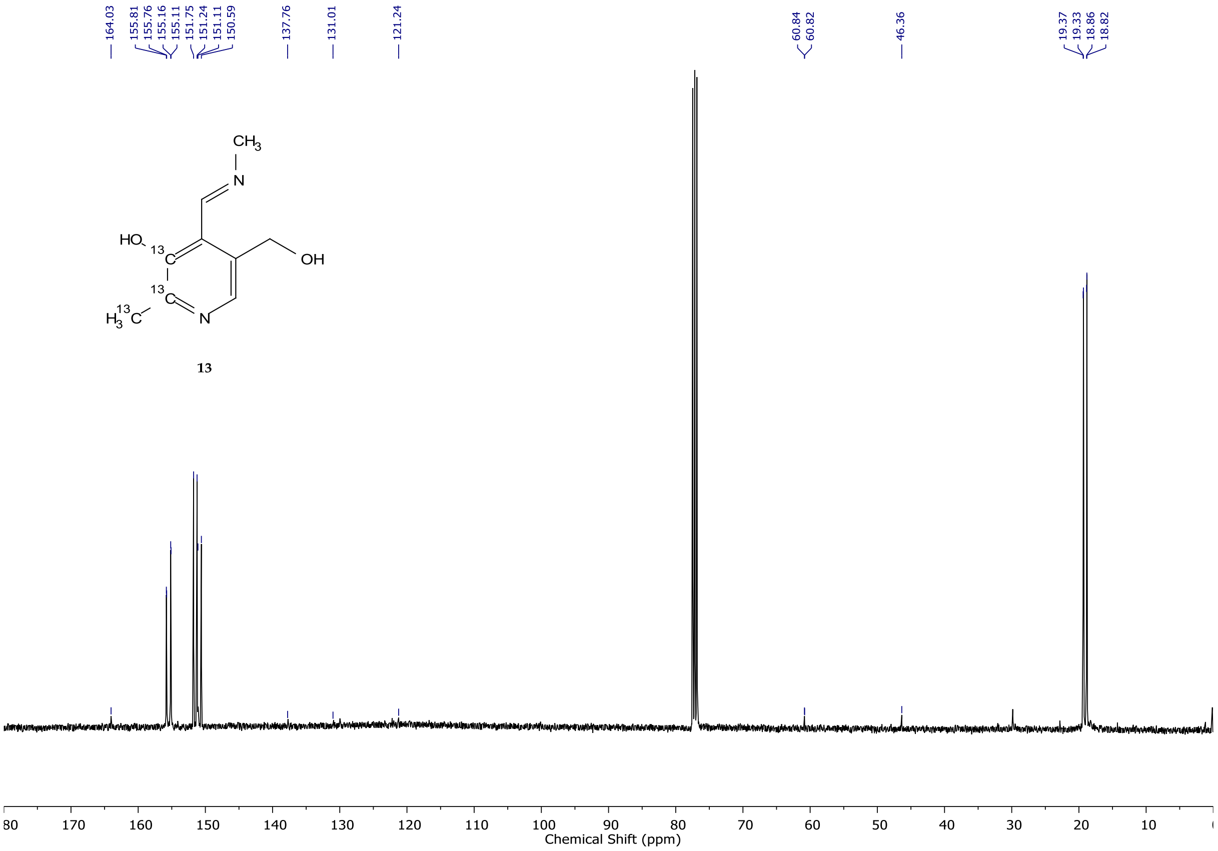

S18. ^{13}C spectrum of ($^{13}C_3$)*N*-(pyridoxylidene)methylamine

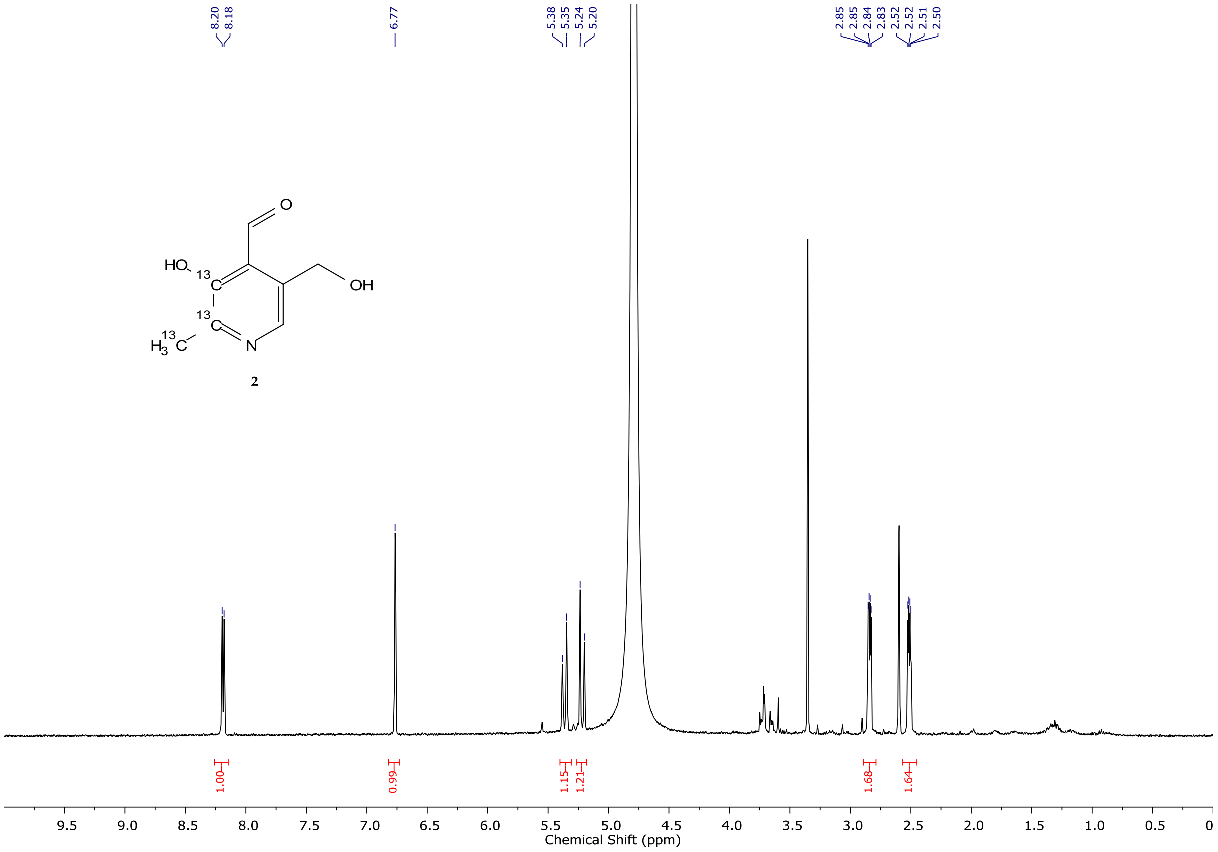

S19. ^{1}H spectrum of ($^{13}C_3$)pyridoxal

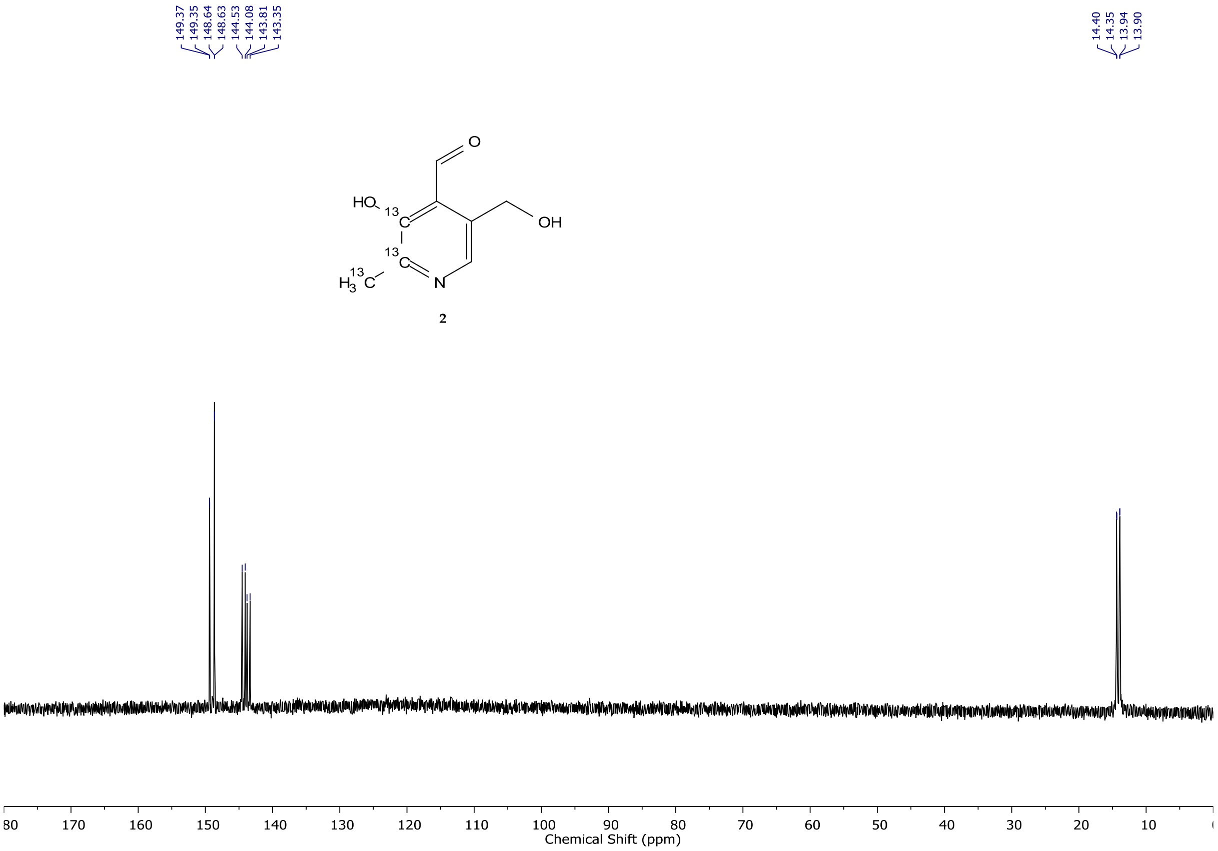

S20. ^{13}C spectrum of ($^{13}C_3$)pyridoxal

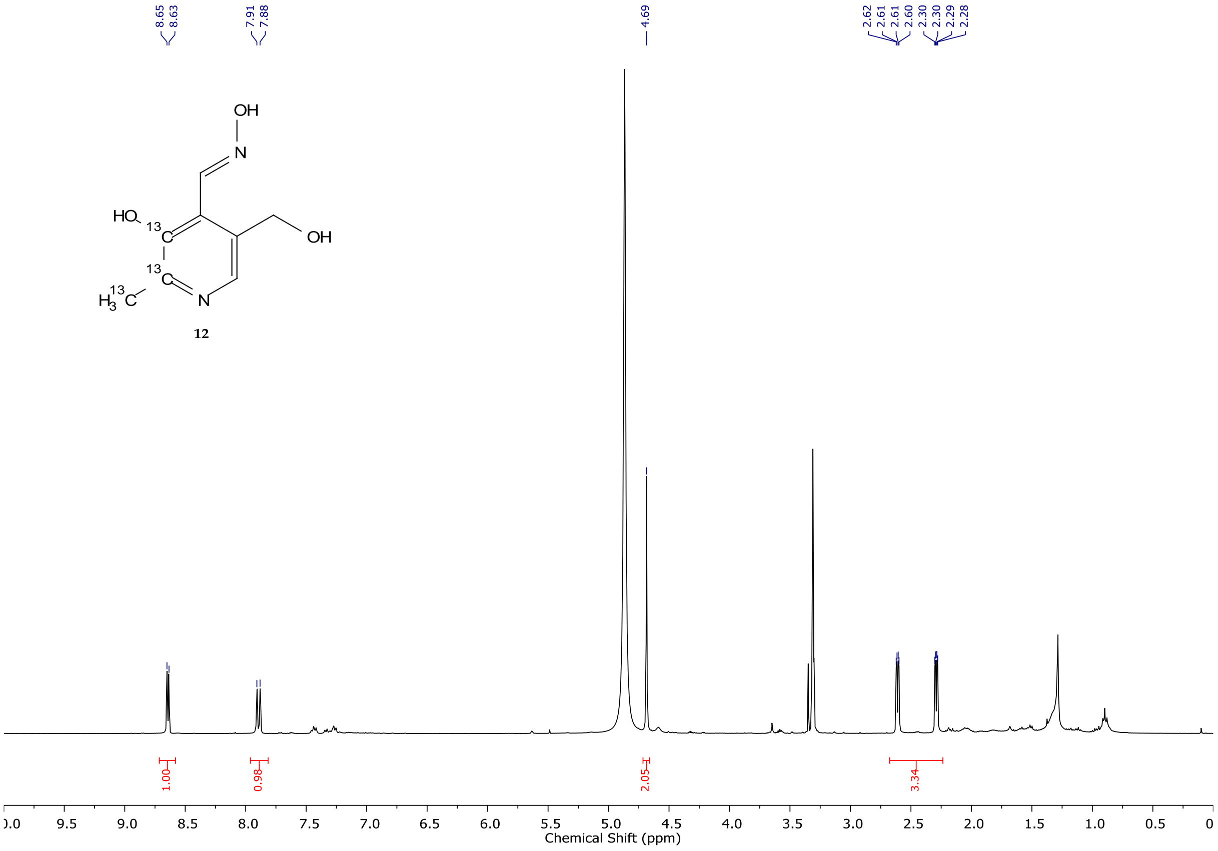

S21. ^{1}H spectrum of ($^{13}C_3$)*N*-(pyridoxylidene)hydroxylamine

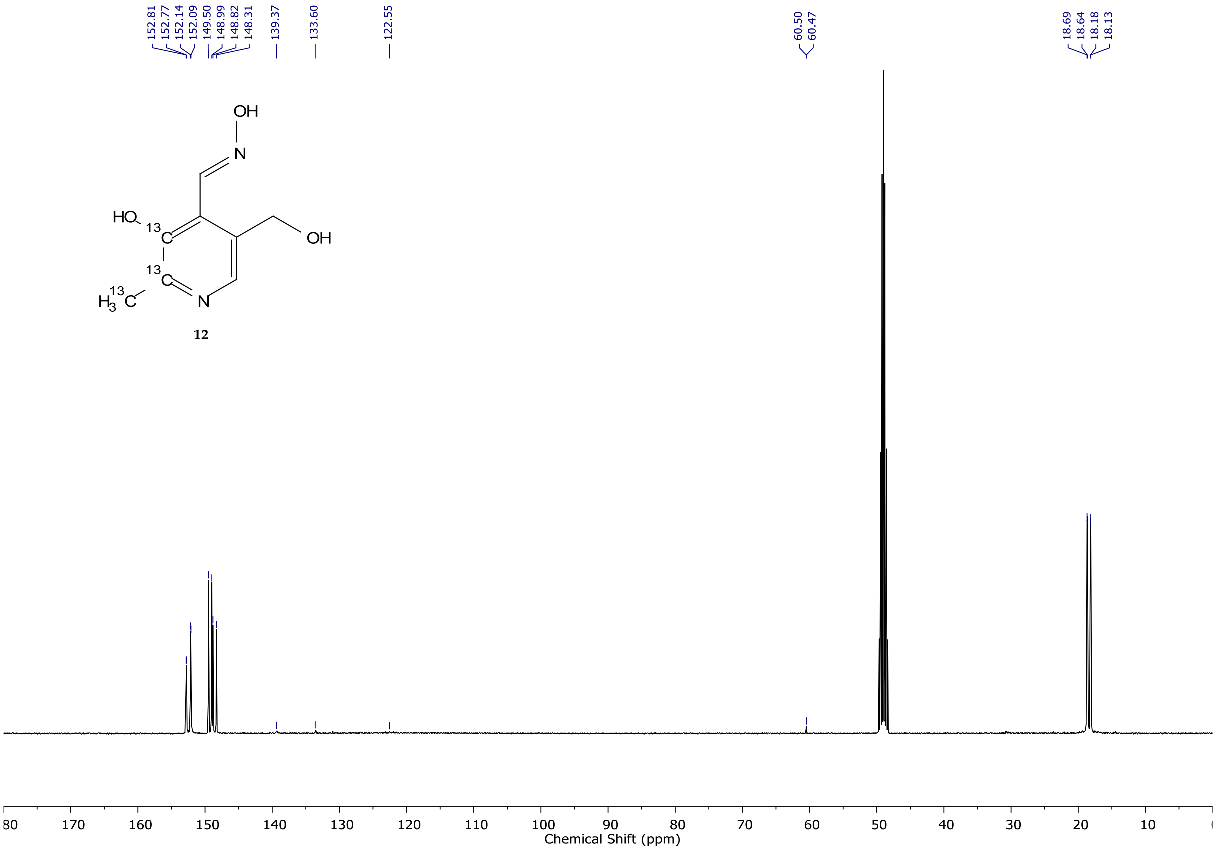

S22. ^{13}C spectrum of ($^{13}C_3$)*N*-(pyridoxylidene)hydroxylamine

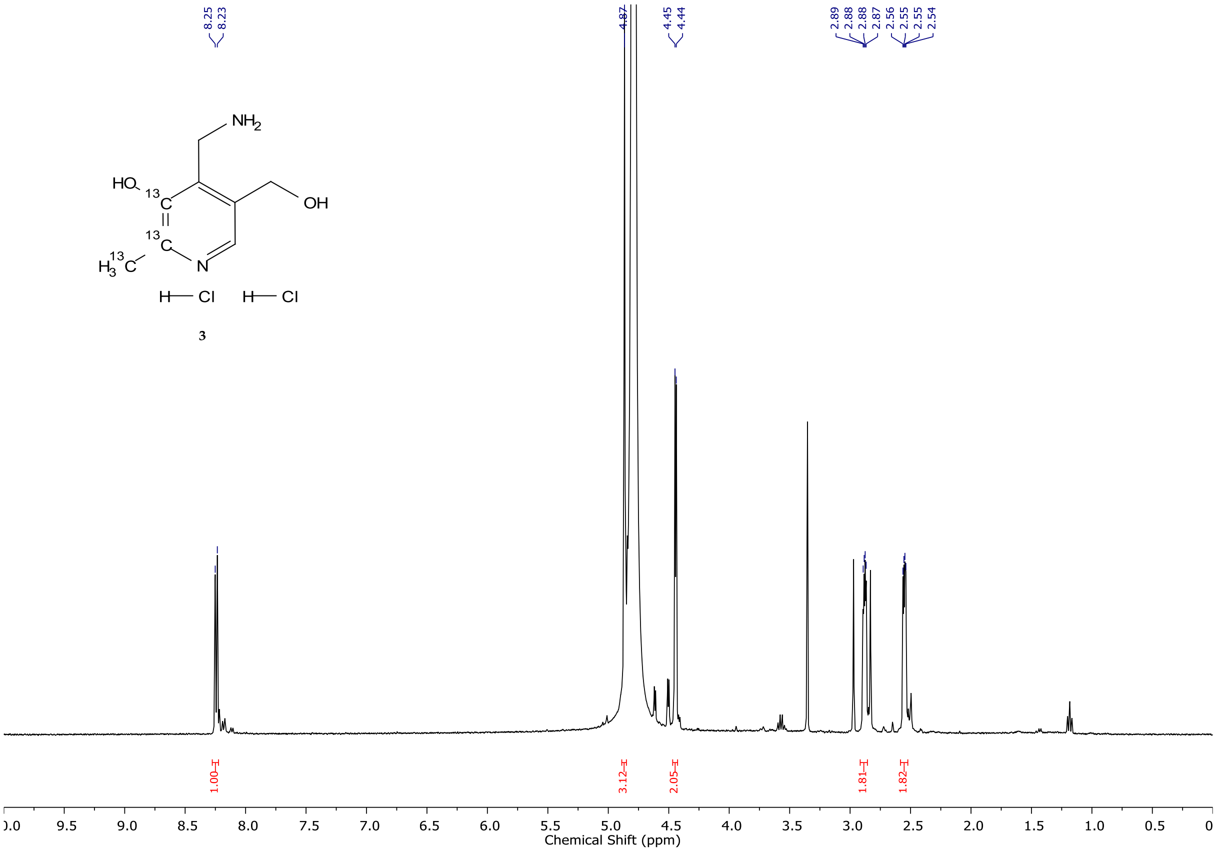

S23. ^{1}H spectrum of ($^{13}C_3$)pyridoxamine

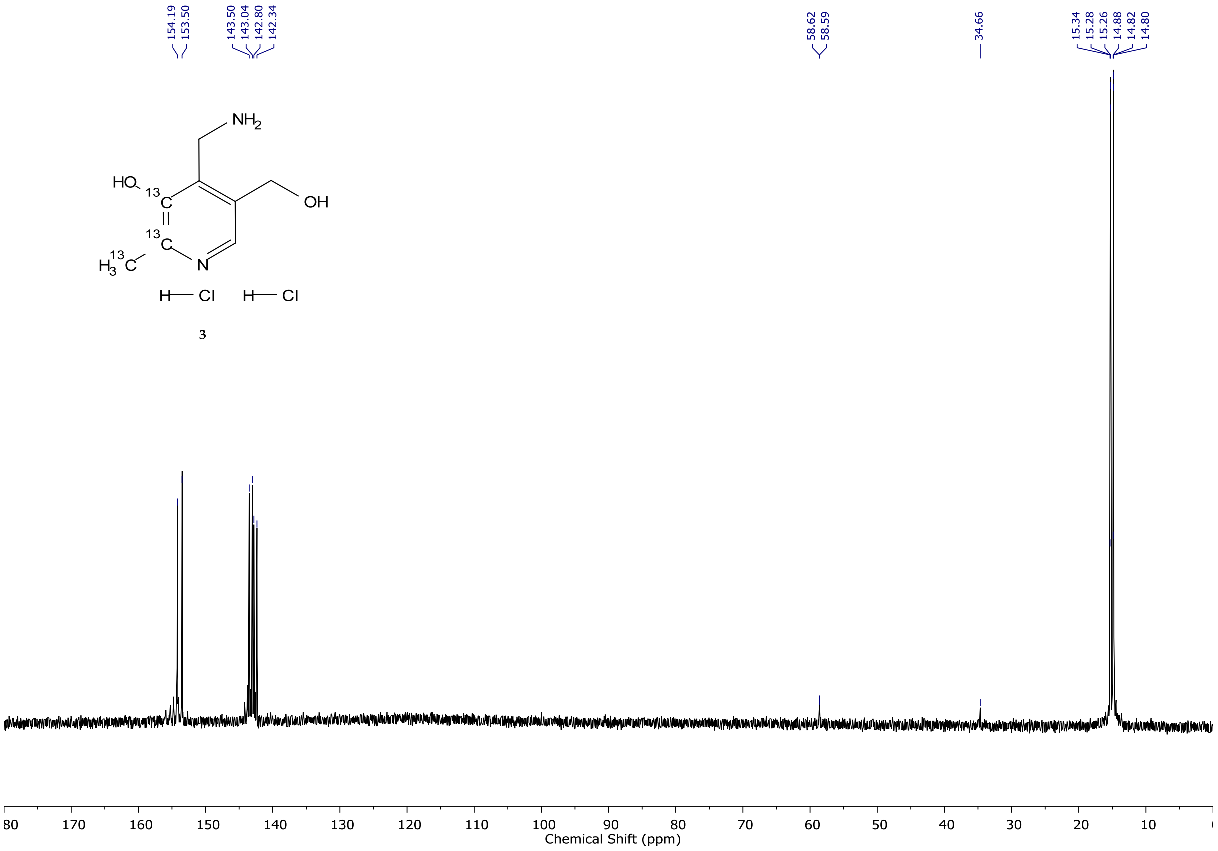

S24. ^{13}C spectrum of ($^{13}C_3$)pyridoxamine
